# Single-cell imaging of ERK and Akt activation dynamics and heterogeneity induced by G-protein-coupled receptors

**DOI:** 10.1242/jcs.259685

**Published:** 2022-03-11

**Authors:** Sergei Chavez-Abiega, Max L. B. Grönloh, Theodorus W. J. Gadella, Frank J. Bruggeman, Joachim Goedhart

**Affiliations:** 1 Swammerdam Institute for Life Sciences, Section of Molecular Cytology, van Leeuwenhoek Centre for Advanced Microscopy, University of Amsterdam, Amsterdam, NL-1098XH, The Netherland; 2 Systems Biology Lab/AIMMS, Vrije Universiteit Amsterdam, Amsterdam, NL-1081HZ, The Netherlands

**Keywords:** GPCR, Biosensor, Fluorescence imaging, Image analysis, Kinase, Signaling

## Abstract

Kinases play key roles in signaling networks that are activated by G-protein-coupled receptors (GPCRs). Kinase activities are generally inferred from cell lysates, hiding cell-to-cell variability. To study the dynamics and heterogeneity of ERK and Akt proteins, we employed high-content biosensor imaging with kinase translocation reporters. The kinases were activated with GPCR ligands. We observed ligand concentration-dependent response kinetics to histamine, α2-adrenergic and S1P receptor stimulation. By using G-protein inhibitors, we observed that Gq mediated the ERK and Akt responses to histamine. In contrast, Gi was necessary for ERK and Akt activation in response to α2-adrenergic receptor activation. ERK and Akt were also strongly activated by S1P, showing high heterogeneity at the single-cell level, especially for ERK. Cluster analysis of time series derived from 68,000 cells obtained under the different conditions revealed several distinct populations of cells that display similar response dynamics. ERK response dynamics to S1P showed high heterogeneity, which was reduced by the inhibition of Gi. To conclude, we have set up an imaging and analysis strategy that reveals substantial cell-to-cell heterogeneity in kinase activity driven by GPCRs.

## Abstract (precis)

**Summary:** High-content biosensor imaging with kinase translocation reporters reveals substantial cell-to-cell heterogeneity in kinase activity driven by G-protein-coupled receptors.

## INTRODUCTION

There are over 500 kinases encoded by the human genome, playing a fundamental role in regulating key biological processes within cells (Manning et al., 2002). Kinases are major drug targets for oncology, with many approved drugs for the treatment of several breast and lung cancer types (Bhullar et al., 2018). Kinases can either phosphorylate serine/threonine residues or tyrosine, or – in some cases – both. The activity of kinases is regulated by events such as ligand binding or phosphorylation by other kinases (Cheng et al., 2011).

G-protein-coupled receptor (GPCR)-mediated signaling pathways involve many different kinases. The best-characterized and studied kinases are PKA (also known as PRKA), PKC (also known as PRKC) and Akt (or PKB) proteins from the AGC family, and the mitogen-activated protein kinases (MAPKs) ERK, p38 (also known as MAPK11) and JNK. The activity of kinases such as PKA or PKC can often be tracked back to specific heterotrimeric G-protein families. For instance, the relative activities of both Gα_s_ and Gα_i_ determine the cAMP levels in the cytosol (Sadana and Dessauer, 2009), and cAMP modulates PKA activity by binding to its inhibitory domain (Taylor et al., 1990). Similarly, PKC is usually activated by increased levels of DAG and Ca^2+^, which occurs as result of PLCβ activation by Gq (Mizuno and Itoh, 2009). In contrast, the activity of kinases such as Akt or MAPKs is more downstream of the G-protein-coupled receptor and, therefore, determined by different G proteins and pathways. The classic downstream effector of Gq is PKC, which can activate ERK. On the other hand, it is not evident how Gq would affect Akt. The molecular network that connects the activity of Gi with kinases is also not so clear. In addition, other components involved in GPCR signaling, i.e. the β-arrestins that are traditionally considered exclusively as mediators of receptor internalization, can activate the MAPK ERK (Luttrell et al., 2001).

Traditionally, kinase activities are inferred from cell lysates, hiding the heterogeneity of the individual cellular responses to extracellular stimuli. With the advent of genetically encoded biosensors, individual cells can be tracked over time. Several fluorescence-based biosensors are available that report kinase activity, each with different designs, fluorophores, read-outs, dynamic ranges and sensitivities (Lee et al., 2020). We decided to use kinase translocation reporters (KTRs) because of the flexibility in the choice of the fluorophore and because they use a single channel (Regot et al., 2014). KTRs suitable for monitoring MAPKs and Akt have been described (Maryu et al., 2018; Miura et al., 2018). Because aberrant behavior of ERK and Akt is found across cancer types, these kinases are heavily investigated as potential therapeutic targets (Cao et al., 2019).

Single-cell studies on GPCR signaling pathways are still scarce, and the majority of studies on ERK activity in single cells are restricted to the study of growth factors. ERK activation kinetics is known to be very dynamic and to vary greatly between growth factors and concentrations (Sampattavanich et al., 2018). Such studies indicate that cell subpopulations can be identified on the basis of single-cell responses. Therefore, we decided to (1) examine whether KTRs are sufficiently sensitive to detect activation of endogenous GPCRs, and (2) to study the contribution of the Gq and Gi protein families to the activities of the kinases ERK and Akt in single cells.

## RESULTS

### Establishing a cell line for detection of ERK and Akt activation with translocation reporters

**Fig. 1.**
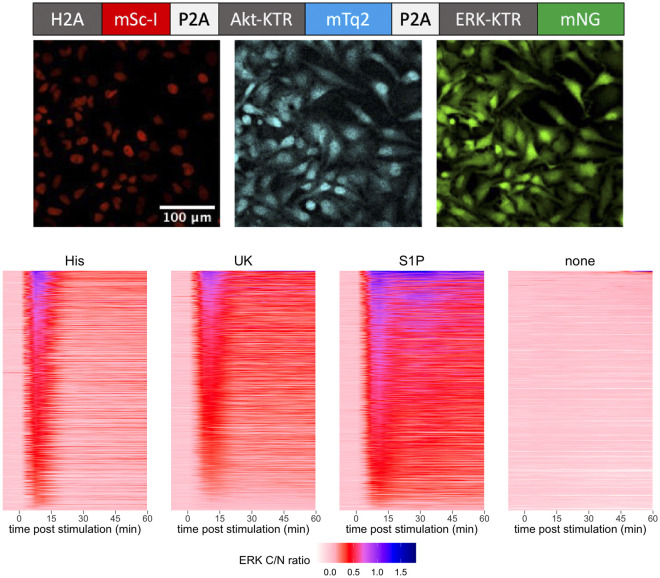
**Construction and application of a HeLa cell line that expresses fluorescent proteins that visualize the nucleus, Akt activity and ERK activity to report on G-protein-coupled receptor activation.** The top panel shows a schematic drawing of the open reading frame of the construct with histone 2A (H2A) tagged with mScarlet-I (mSc-I), the Akt kinase translocation reporter (Akt-KTR) tagged with mTurquoise (mTq2) and the ERK kinase translocation reporter (ERK-KTR) tagged with mNeonGreen (mNG). The P2A sequences ensure separation of the proteins. The middle panel shows HeLa cells expressing the construct. From left to right: nuclear marker, Akt-KTR and ERK-KTR in red, cyan and green, respectively. The lower panel shows time-lapse ERK responses to maximum ligand stimulatory concentrations, where each row reflects a single cell. HeLa cells were treated with 100 µM histamine (His), 100 pM UK 14304 (UK), 1300 nM sphingosine-1-phosphate (S1P) or no ligand (none). The ligand was added at *t*=0 and remained present. The ERK cytoplasmic to nuclear intensity (C/N) ratio is presented as a false color and reflects the cytoplasmic over nuclear ratio of the ERK-KTR, normalized by subtracting the average from the two time points prior to stimulation. For each ligand, the data correspond to at least three biological replicates, which are combined and sorted according to their integrated response.

To investigate the relationship between heterotrimeric G proteins and the activities of ERK and Akt in single cells, we employed KTRs. To detect Akt and ERK, we used Akt-FoxO3a-KTR (Maryu et al., 2016) tagged with mTurquoise2 (mTq2) (Goedhart et al., 2012), and ERK-KTR (Regot et al., 2014) fused with mNeonGreen (mNG) (Shaner et al., 2013). To facilitate the identification of nuclei, we added a histone-tagged mScarlet-I (mScI) (Bindels et al., 2016). The open reading frames of the three components were connected with P2A sequences, which ensures quantitative co-expression of the three proteins from a single open reading frame. The plasmid is named HSATEN (histone-Scarlet-I | Akt-KTR-mTurquoise2 | ERK-KTR-mNeonGreen). A scheme of the open reading frame of HSATEN and HeLa cells expressing it is shown in Fig. 1.

We used the PiggyBac transposon system (Matasci et al., 2011) to generate cells that stably express the triple color reporter, and used fluorescence-activated cell sorting (FACS) to isolate single cells and obtain monoclonal populations. As can be seen in Fig. S1, ∼60% of the cells were positive for mNG and mScI. Using the green fluorescence intensity levels, we sorted cells with intermediate levels of fluorescence into four pools. Next, we characterized the KTR response to fetal bovine serum (FBS), which strongly activates growth factor signaling and kinase activity.

To quantitatively compare the responses, we set up an analysis pipeline that quantifies the ratio of the cytoplasmic to nuclear intensity (C/N) of single cells, reflecting the kinase activity (Maryu et al., 2016). The pipeline uses FIJI (Schindelin et al., 2012) for background correction, CellProfiler (McQuin et al., 2018) for segmentation, and the R programming language (https://www.r-project.org/) for processing and visualizing the data. Fig. S2 shows the different steps of the analysis procedure. The scripts and fully reproducible instructions are available at https://github.com/JoachimGoedhart/Nuclear-translocation-analysis. This analysis pipeline is used for all data presented in the paper.

Based on the KTR responses, we decided to continue with pool 3. To examine whether the ERK and Akt basal levels could be reduce by serum starvation, we replaced the growth medium with serum-free imaging medium and followed the C/N ratio over time. A reduction in the C/N ratio was observed and this reached a plateau after ∼100 min (Fig. S3A). All of the following experiments were performed ∼2 h after replacing the medium to reduce the basal activity of ERK and Akt.

Next, we examined the effect of the MEK inhibitor PD 0325901. Pre-incubation with the inhibitor for 20 min blocked the response of the ERK-KTR to FBS, but not that of the Akt-KTR (Fig. S3B). This supports previous observations (Maryu et al., 2016; Goedhart et al., 2012) that the P2A effectively separates the different components, because the Akt-KTR and ERK-KTR show independent relocation patterns. The pool was used to isolate several clones. Each of the monoclonal cell lines was tested for their response to FBS, and the fluorescence intensity of the biosensors was quantified (Fig. S4, Table S1). A single clone was selected and used for the remainder of our studies.

### Activation dynamics of ERK after GPCR activation

We selected three GPCR families, based on their capacity to activate different families of heterotrimeric G proteins and their expression in HeLa cells. We selected histamine receptors (HRs) (Jain et al., 2016; Meisenberg et al., 2015) and sphingosine-1-phosphate receptors (S1PRs) (Gandy et al., 2013), for which we used the respective endogenous ligands histamine and S1P. We also selected α_2_-adrenergic receptors (α2ARs) (Gibson and Gilman, 2006) and used UK 14304 (UK), also called brimonidine, a widely used full agonist with very high potency and selectivity (Kurko et al., 2014).

To examine the activation of ERK by the three different GPCRs, we added a saturating concentration of agonist to the HeLa cell line expressing the KTR reporter. All three agonists were capable of inducing an increase in ERK activation as measured by an increased C/N ratio. The responses were transient and showed considerable heterogeneity in amplitude (Fig. 1).

### Concentration–response curves of ERK activation

Next, we examined the effect of different concentrations of agonists. Histamine stimulation caused a transient increase in ERK activity from concentrations as low as 0.19 µM, as shown in Fig. S5A. The maximum activity was concentration dependent and was reached ∼10 min post-stimulation. Similarly, UK addition led to a rapid increase in ERK activity and reached a transient maximum 10–15 min post-stimulation, after which it decreased to reach a plateau 30 min later (Fig. S5B). The increase was observed with concentrations as low as 0.41 pM. In contrast to histamine and UK, S1P showed a more complex pattern with peaks at different time points, depending on the concentration of the agonist (Fig. S5C). Overall, the ERK activity was concentration dependent for all three agonists, with considerable heterogeneity at all of the tested concentrations.

**Fig. 2.**
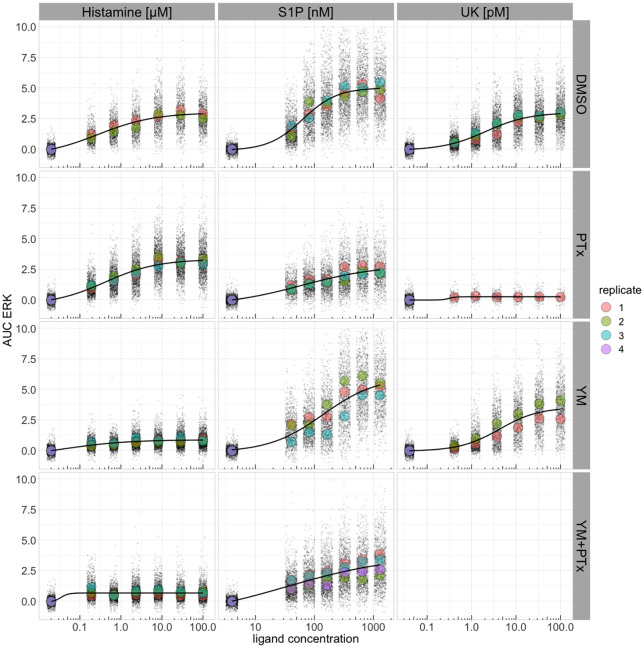
**Concentration**–**response curves for ERK activity under different conditions.** The area under the curve (AUC) was used as the measure of response. The AUC was calculated as the sum of normalized C/N ratios from time points 9–18, corresponding to 7–38.5 min post-stimulation. The data were fitted with a four-parameter logistic equation, using the average of the average ERK AUC per biological replicate. Biological replicates are represented by different colors and their average is shown as a large dot. DMSO, dimethyl sulfoxide; PTx, pertussis toxin; YM, YM-254890.

We used the ERK-KTR data to fit concentration–response curves for ERK activity using the area under the curve (AUC) as the measure of the response, which we calculated as the sum of C/N ratios between 7 and 38.5 min post-stimulation. For each biological replicate at every concentration, we calculated the average AUC, indicated by a large dot in Fig. 2. The average of the biological replicates was used to fit the curve, and the results are shown in Fig. 2 and Table S2. The half-maximal effective concentration (EC50) values for histamine, S1P and UK were 0.3 µM, 64 nM and 2.5 pM, respectively.

### Effect of inhibiting heterotrimeric G proteins on ERK and Akt activation

To examine the role of heterotrimeric G proteins in the activation of ERK and Akt, we used YM-254890 (YM) to inhibit Gq and pertussis toxin (PTx) to inhibit Gi (Campbell and Smrcka, 2018). After inhibitor treatment, we stimulated the cells with histamine, S1P or UK in a range of concentrations. The dynamics of the responses are reported in Fig. S5. The AUC was used to construct concentration–response curves, and these are depicted in Fig. 2. We note that YM, which targets Gq, inhibits the ERK response by histamine, whereas the response to UK is largely inhibited by PTx, which interferes with Gi signaling. The response to S1P is hardly affected by YM, but the amplitude is reduced by PTx.

**Fig. 3.**
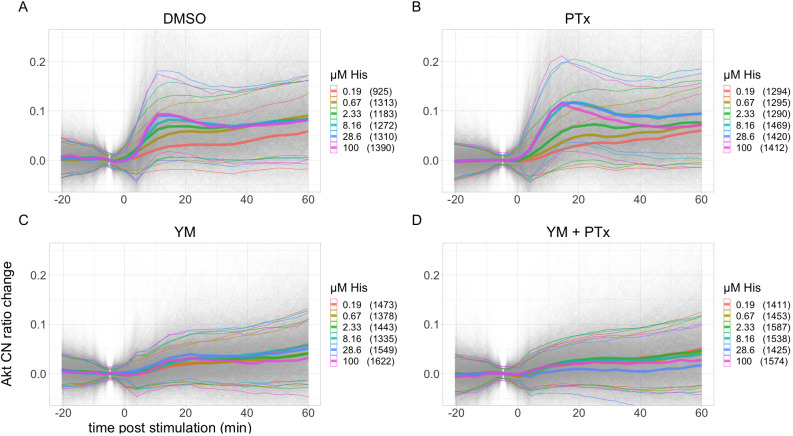
**Akt responses to different concentrations of histamine and the effect of Gq and Gi inhibition.** (A) No inhibitor (DMSO). (B) Gq inhibition (YM). (C) Gi inhibition (PTx). (D) Combined Gq and Gi inhibition (YM+PTx). Akt C/N ratio change is calculated by subtracting the average from the two time points prior to stimulation. Each panel shows combined data from at least three biological replicates. Gray lines represent single-cell traces. Thick colored lines show the mean and thin colored lines the s.d. for each ligand concentration. Numbers of cells are shown between brackets.

Next, we examined the responses of Akt, which is simultaneously measured. The Akt responses were noisier due to lower amplitudes. Fig. 3 shows the Akt responses to histamine. In the absence of inhibitors, the Akt activation is partially transient, with the response peaking 10 min post-stimulation and decreasing in the following 25 min to reach baseline levels (Fig. 3A). Gi inhibition appears to cause a small increase in maximum activity and possibly a short delay in time of maximum activity, as shown in Fig. 3B. Inhibition of Gq (Fig. 3C) decreases the maximum activity up to ∼70%, and simultaneous inhibition of Gq and Gi causes a decrease in the responses by up to ∼90%, as shown in Fig. 3D. These Akt amplitudes and effects of inhibitors are largely similar to those observed for ERK.

### ERK and Akt activities are correlated

**Fig. 4.**
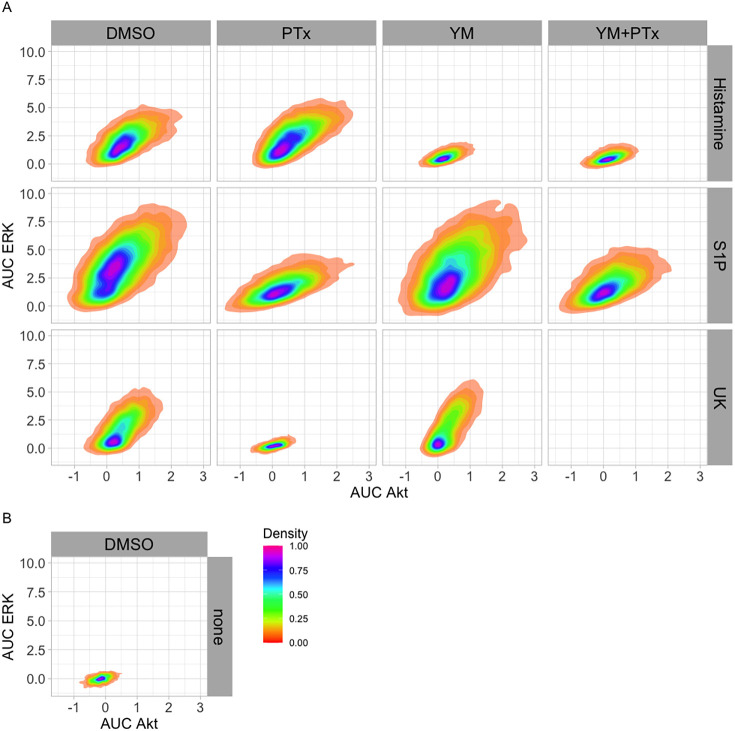
**Activity of ERK versus activity of Akt per cell.** The AUC is used as the measure of response and was calculated as the sum of normalized C/N ratios from the time points 9–18, corresponding to 7–38.5 min post-stimulation. Saturating concentrations of the ligands were used. For each cell, the AUC of Akt was plotted against the AUC of ERK, and the data from all biological replicates per condition are shown.

It is striking that Gq inhibition has a similar inhibitory effect on Akt and ERK activity when cells are treated with histamine. To examine the correlation between ERK and Akt activity in more detail, we calculated the integrated response (AUC) for ERK and Akt in every cell for the different treatments. By plotting the ERK versus Akt activity, the relationship between both activities can be visualized. As can be inferred from Fig. 4, there is a moderate positive correlation between both kinase activities for each ligand. In conditions in which G-protein inhibition drastically affects the activity of the kinases, such as YM for histamine and PTx for S1P, the ERK responses are more strongly reduced than the Akt responses. Finally, we note that, for S1P, the Akt activity is hardly or not reduced in the presence of inhibitors.

### Basal kinase activity does not affect the response amplitude

The measured single-cell kinase activities within an experimental condition, or even within a biological replicate, exhibit considerable heterogeneity. This can be clearly observed in the data shown in Figs 1–4. A possible explanation for the observed heterogeneity is that differences in basal kinase activity affect how the cells respond to the stimulus.

**Fig. 5.**
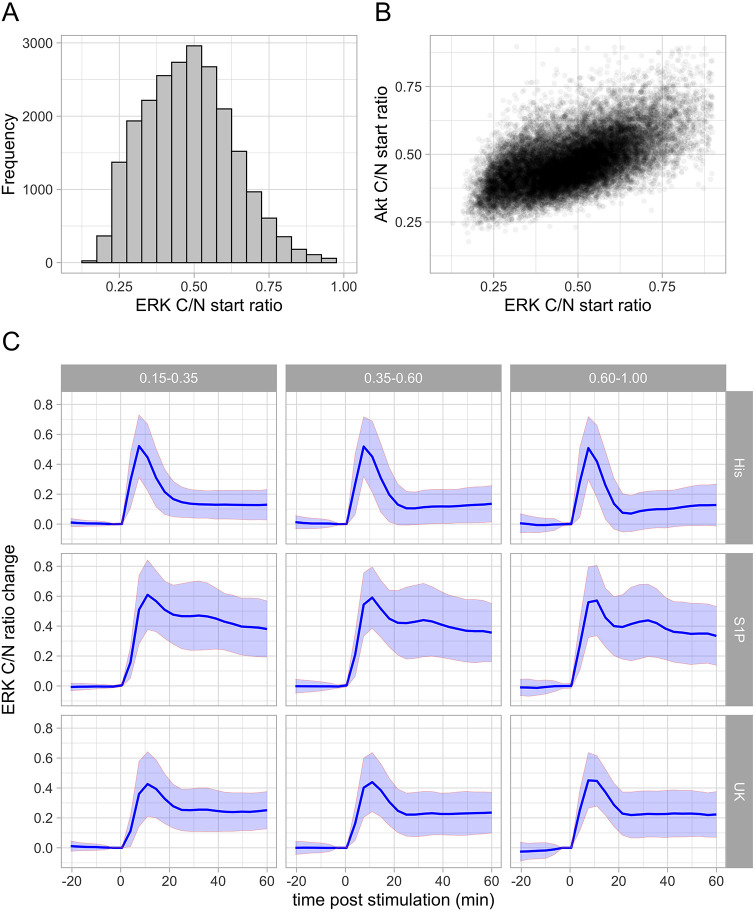
**Distribution of start C/N ratios and effect on the ERK response.** (A) Frequency of the average C/N ratios of ERK prior to ligand stimulation, using the data from single cells from all the experiments. (B) Relationship of the resting ERK C/N ratios and the resting Akt C/N ratios. (C) The data for the ERK responses at the maximum concentration of each of the ligands were grouped according to the start ratio, as indicated in the labels on top of the graphs. The ‘low’ pool had a start ratio of 0.15–0.35, the middle pool a start ratio of 0.35–0.6 and the ‘high’ pool a start ratio of 0.6–1.0. The ERK C/N ratio change was normalized by subtracting the average of the two time points prior to stimulation. The line shows the average and the ribbon shows the s.d.

The information on basal kinase activity is lost when the data are normalized to set the initial C/N ratio to unity. To examine how the initial C/N ratio affects the response dynamics, we looked at the original, non-normalized data. Fig. 5A shows the variability among the C/N ratios for each KTR before ligand stimulation. For ERK, these start C/N ratios are spread evenly between 0.20 and 0.75. In Fig. 5B, the ERK and Akt C/N ratios from individual cells are plotted, showing a weak correlation and start C/N ratios for Akt mostly between 0.30 and 0.65.

To examine whether the start C/N ratios, which reflect basal kinase activity, have an effect on the absolute C/N ratio changes, we decided to split each of the three datasets (without inhibitors) into three groups that represented relatively low, medium and high start ratios. Fig. 5C shows the results for ERK. Overall, cells with different start ratios show comparable curve shapes and maximum activity for the three ligands. For the lowest concentrations, there appears to be a trend in which the ERK maximum activity increases slightly with the start ratio, but the differences are relatively small.

To conclude, our data show that the absolute changes in C/N ratios are hardly or not affected by the start C/N ratios. This suggests that the measured biosensor responses are not saturated in our experiments and that we can capture the entire range of kinase activities.

### Clustering reveals different kinase responses to GPCR activation

To gain more insight into the heterogeneity and possible patterns in the response, we turned to cluster analysis. Clustering simplifies the data by defining different categories that group mathematically similar responses. This method had previously been used to examine the response of fluorescence resonance energy transfer (FRET) biosensors (Kuchenov et al., 2016) and KTRs (Gagliardi et al., 2021). First, we used a subset of the data to explore the optimal clustering method and to find the optimal number of clusters.

From our data, it is clear that there are major differences between the quantified ERK and Akt responses. First, the dynamic range of the ERK responses to the ligands is approximately three to four times bigger than that of the Akt responses. Second, the ERK responses display various different curve shapes, whereas the Akt responses vary almost exclusively in amplitude. Third, owing to the low dynamic range, small variations in the focal plane during imaging can have a significant effect on the Akt ratios. For these reasons, we decided to evaluate three to five clusters for Akt, and eight to ten clusters for ERK. We consider that these cluster numbers capture most of the variability in the data, without complicating interpretation of the results, providing high-quality meaningful information. In addition, we chose to use the C/N ratio changes between 7 and 38.5 min post-stimulation, as this time range contains most of the information.

Owing to popularity for trajectory analysis and access to clustering programming packages, we chose to use k-means clustering and hierarchical clustering. After applying the different clustering methods to a subset of the combined data from all ligands and conditions, we used several metrics to assess and compare the quality of the clustering methods. The advantage of considering several metrics is that we reduce the risk of picking a cluster number that may be favored by a single indicator, but not by the rest. For each of the metrics, the higher the output, the better the quality of the result.

Fig. S6 shows the metrics for various cluster numbers for the ERK and Akt data. As can be observed, the multiple metrics do not always show similar trends, which is not surprising given the differences in the ways they are calculated. In order to combine the different metrics to select clustering candidates, we decided to normalize each of the metric values by dividing it by the highest value among all the 15 combinations. Then, for each combination (cluster method and number), we added the values from all metrics, and the results are shown in Table S3. From these, we picked two combinations per kinase, shown in blue, based on a higher score and lower number of clusters. Finally, we generated two plots to inspect the selected clustering approaches. First, we plotted the distribution of the clusters among the negative controls (no ligand added) and the experiments with the highest ligand concentrations. Second, we plotted the trajectories per cluster, using all 15,000 cells. These plots are shown in Fig. S7. Both algorithms yielded similar results, and we decided to use eight clusters for ERK with the Manhattan distance and Ward2 linkage method. For Akt, we chose three clusters based on the Euclidean distance with Ward2 linkage.

**Fig. 6.**
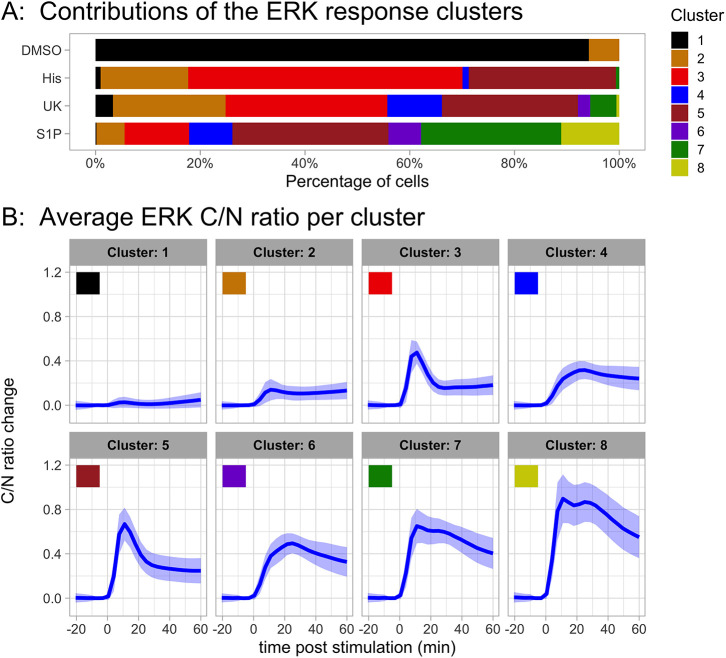
**Results of clustering all data for the ERK responses.** The selected method has eight clusters and uses Manhattan distance and the Ward2 linkage method. It was applied to all the cells from the combined experiments with different ligands, concentrations, conditions and negative controls. (A) Cluster distribution of responses in a control and in the condition of maximal ligand concentration. The control reflects addition of medium instead of ligand. (B) Average trajectory and frequency of each cluster. Per cluster, the lines represent the average trajectory and the ribbon the s.d.

Once we had selected the clustering method and optimal number of clusters, we applied it to the combined data from all ligands and inhibitory conditions (∼68,000 cells). Fig. 6 shows eight distinct response patterns for ERK activation, including no and low responses (cluster 1 and 2), transient responses (cluster 3 and 5) and different patterns of a more sustained response (cluster 4, 6, 7 and 8).

Our initial qualitative judgement that the response to histamine and UK is similar is also quantitatively supported by the graph in Fig. 6A that shows the contribution of each cluster to a treatment. A transient response dominates for these agonists. In contrast, the response to S1P is very heterogeneous, with contributions of cells that show transient ERK activity and cells that show sustained activity. The biphasic ERK activation pattern, which is specific for stimulation with S1P, is reflected by clusters 7 and 8.

**Fig. 7.**
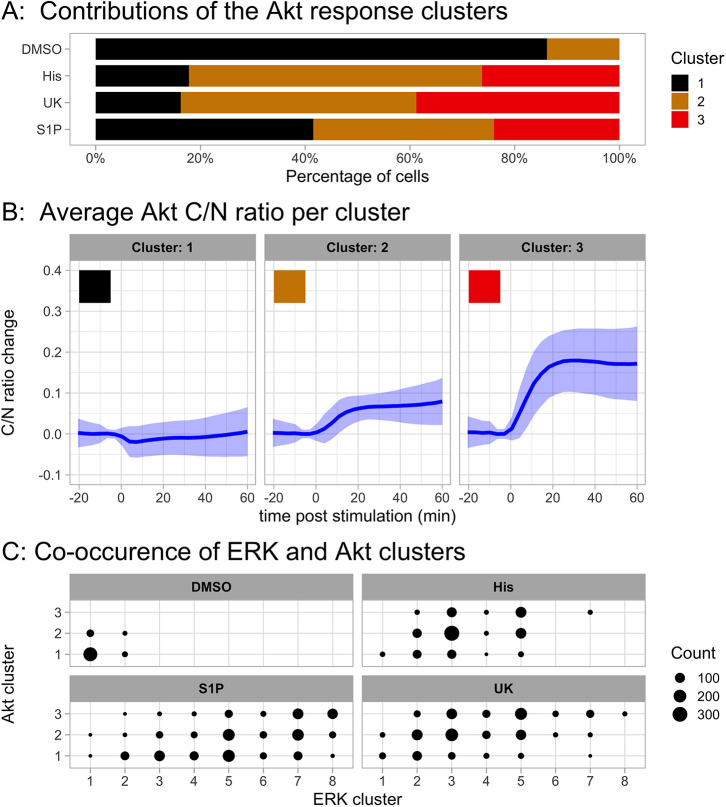
**Results of clustering all data for the Akt responses.** The selected method has three clusters and uses Euclidean distance and the Ward2 linkage method. It was applied to all the cells from the combined experiments with different ligands, concentrations, conditions and negative controls. (A) Cluster distribution of responses in a control and in the condition of maximal ligand concentration. The control reflects addition of medium instead of ligand. (B) Average trajectory and frequency of each cluster. Per cluster, the lines represent the average trajectory and the ribbon the s.d. (C) Co-occurrence of the ERK and Akt clusters for the control condition and for each of the three ligands at maximal concentration.

The cluster analysis for Akt is shown in Fig. 7. The responses are grouped in three patterns, one of non-responding cells and two with sustained responses, differing in amplitude. The activation of Akt is remarkably similar between the different treatments.

In Fig. 7C, the co-occurrence of ERK and Akt clusters is depicted. Also, in this plot, there is similarity between the responses to the ligands histamine and UK, with a high co-occurrence of transient ERK activation (clusters 3 and 5) with a sustained Akt response (clusters 2 and 3). The response to S1P shows again a larger heterogeneity.

**Fig. 8.**
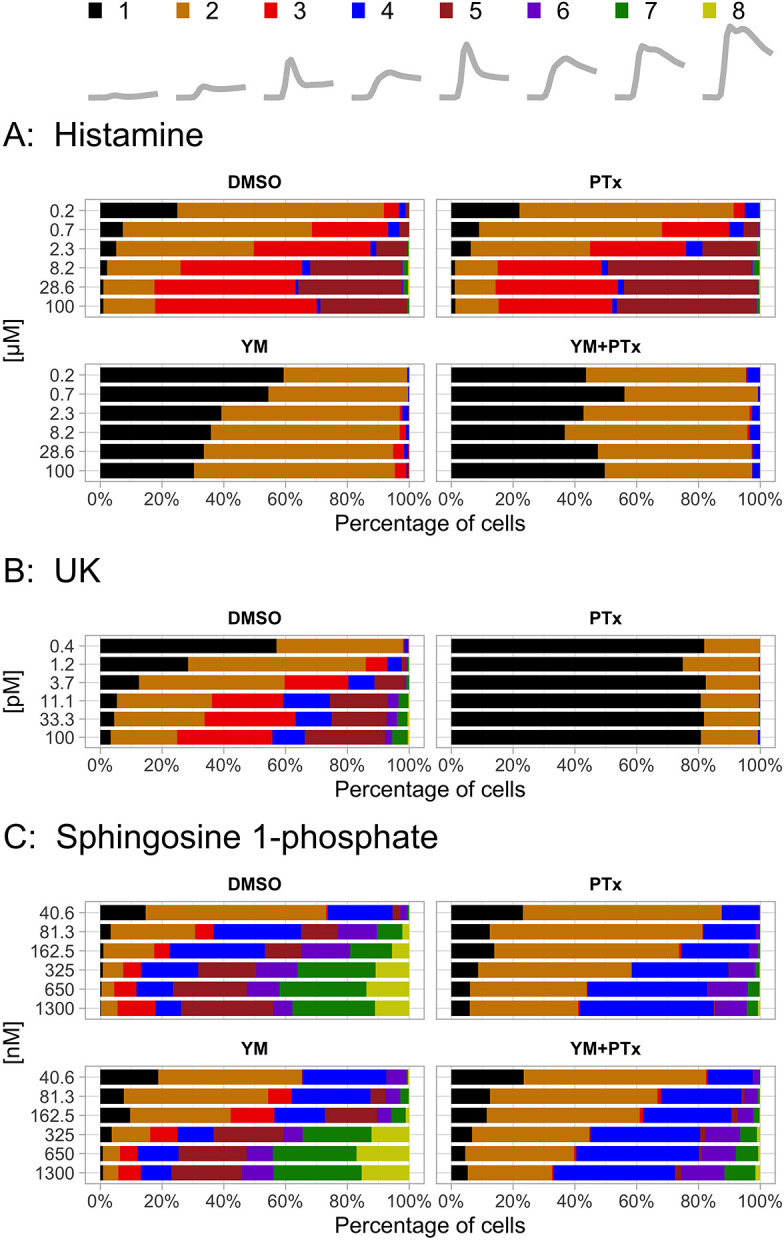
**Cluster distribution of ERK responses at different concentrations per ligand.** The temporal profile and the corresponding color code of each cluster (repeated from Fig. 6B) is indicated as a key at the top of the figure. (A–C) For each ligand, histamine (A), UK 14304 (B) and sphingosine-1-phosphate (C), the relative contribution of the clusters is shown for the different treatments: no inhibitor (DMSO), Gq inhibition (YM), Gi inhibition (PTx), and combined Gq and Gi inhibition (YM+PTx).

Because the ERK activation shows the largest heterogeneity, we examined the effect of heterotrimeric G-protein inhibition. For each of the conditions, we display the relative contribution of each of the different patterns. The results are depicted in Fig. 8 and Fig. S8A. The results for Akt are shown in Fig. S8B.

The fraction of non-responding cells (cluster 1) systematically decreases when the ligand concentrations of histamine, UK or S1P are increased. Although the inhibitors are effective in some combinations, i.e. YM and histamine, PTx and UK, there is still a fraction of cells that respond. This suggests that the ERK activity is not exclusively due to the activation of the corresponding heterotrimeric G protein.

We note that there are hardly any unresponsive cells for S1P concentrations above 81.3 nM. However, there is substantial heterogeneity in the responses above this concentration. At least six different response patterns can be discerned. Surprisingly, the heterogeneity is strongly reduced when Gi signaling is inhibited by PTx. The rapid rise in ERK activation (as observed for stimulation by histamine and UK) is abolished and a delayed and more sustained response is the result. The effect of YM on the ERK activity is weak. To summarize, cluster analysis reveals the contribution of different ERK activation patterns and the palette of patterns can be profoundly changed by inhibition of heterotrimeric G proteins.

## DISCUSSION

Most studies of kinases activated downstream of GPCR signaling pathways are performed using biochemical assays on cell populations. These methods cannot measure the dynamics in individual cells and detect the heterogeneity of the individual responses. The recent engineering of fluorescent biosensors that are based on translocation has enabled high-content imaging of kinases such as ERK, Akt, JNK and p38. These reporters have been successfully used to study growth factor signaling in a number of settings and systems (Blum et al., 2019; Ryu et al., 2016). So far, only a couple of studies looked into kinase activation by GPCRs in single cells with KTRs and these studies used overexpressed receptors (Jung et al., 2017; Spinosa et al., 2019).

Here, we use KTRs that report on ERK and Akt (Maryu et al., 2016) to generate monoclonal stable cell lines that can be used for multiplex imaging and demonstrate that the KTRs are sensitive enough to detect activation of endogenous GPCRs. This is in marked contrast to other fluorescent biosensors that, in our hands, typically require an overexpressed receptor for robust responses (van Unen et al., 2016a). Our imaging pipeline enables high-content imaging of the responses, yielding quantitative, dynamic data from thousands of cells. The data were used to generate concentration–response curves for three agonists from the imaging data and to examine the effect of G-protein inhibition. The analysis revealed different dynamics between GPCRs, and the cluster analysis showed differences between subpopulations of cells activated with the same agonist.

Our initial idea was to use KTRs as specific read-outs for heterotrimeric G-protein activity, which is relevant for understanding ligand-biased activation (Kenakin, 2019). This would be achieved when Gq activation is linked to ERK and Gi activation results in Akt activity. We selected three agonists that would activate three different families of GPCRs that are endogenously present in HeLa cells. Histamine is reported to predominantly activate Gq in HeLa cells by the histamine H1 receptor (Pietraszewska-Bogiel and Goedhart, 2019 preprint), and UK activates Gi by α2-adrenergic receptors (van Unen et al., 2016b). Our data with the inhibitors YM and PTx, which are selective for Gq and Gi, respectively, show that the two agonists indeed preferentially activate a single heterotrimeric G-protein class.

Despite the activation of different heterotrimeric G-protein families, the responses of the ERK-KTR to histamine and UK are remarkably similar. Both agonists also show a comparable effect on the amplitude and kinetics of the Akt-KTR. Therefore, our choice of KTRs does not enable the discrimination of signaling through Gi and Gq. The combination of an ERK-KTR and Akt-KTR is not optimal, because their activities are largely correlated and similar for different G-protein classes. Therefore, the measurement of Akt does not add information. Moreover, the Akt response had a relatively poor amplitude.

The situation for S1P is different. S1P can activate a number of different GPCRs, all known to be expressed by HeLa cells as shown in supplemental figure S4A of Gandy et al. (2013). As a consequence, S1P will activate a number of different heterotrimeric G-protein families. We observed that activation of endogenous S1P receptors resulted in a strong, but highly heterogeneous, ERK-KTR response, with two peaks in a population of cells. Both the dynamics and the amplitude varied between populations of cells, and cluster analysis was applied to define eight different patterns (including a flat line for non-responding cells). At least six of these patterns were identified at the higher S1P concentrations. From these data, it is clear that genetically identical cells can respond in a highly heterogeneous manner to a single ligand, which is in line with previous studies (Niepel et al., 2009). Intriguingly, the heterogeneity in ERK dynamics is reduced when Gi signaling is inhibited. When PTx is present, the biphasic response is abolished and the first peak of activation is reduced, suggesting that the initial response is due to Gi signaling. This result demonstrates that, by modulating the palette of heterotrimeric G proteins, the response dynamics are altered, which can be readily identified by cluster analysis. The clustering is a powerful method for the detection of patterns and simplification of large amounts of data. Yet, it should be realized that clustering is mathematical procedure that is not necessarily reflecting the biological processes. One example is the graded response of ERK and Akt activities to ligands, whereas cells are grouped as weak, middle and strong responders. This may be solved by developing and using clustering methods that take the underlying biological processes into account.

To enable better insight into the specific heterotrimeric families that are activated by GPCRs, future studies looking into the response of different KTRs to different heterotrimeric G proteins and agonists are required. There is a translocation reporter, PKA-KTR, which is expected to be specific for Gs (Regot et al., 2014), and there are several KTRs for which selectivity remains to be examined, e.g. p38-KTR and JNK-KTR. In addition, existing proteins that translocate in response to cell stimulation, including MRTF-A, YAP, NF-κB and SMAD, can be examined.

The origins of the observed heterogeneity are unclear at present. We have used a monoclonal cell population, and, therefore, the origin of the heterogeneity is likely to be non-genetic. In addition, we have verified that the differences between cells are not due to saturation of the sensors. Despite the use of monoclonal populations, gene expression is a stochastic process (Sanchez and Golding, 2013), and cellular noise resulting in differences in the relative concentrations of the components involved in the signaling network may lead to the observed differences (Niepel et al., 2009). Moreover, it is unclear what the consequences of this heterogeneity in kinase activities as a result of GPCR activation are. Heterogeneous single-cell response dynamics has previously been linked to differences in physiologically relevant processes such as proliferation (Albeck et al., 2013), metabolic adaptations (Hung et al., 2017), migration (Aoki et al., 2017) and cell fate (Johnson and Toettcher, 2019). Because GPCRs are expressed ubiquitously and participate in many different processes, the implications of this heterogeneity need to be studied in a specific physiological context. Importantly, given the long-term cellular effects of ERK and Akt kinase activities, special attention should be given to changes in gene expression or the cell cycle.

A limitation of our work is that the contribution of G-protein-independent mechanisms for ERK and Akt activation are unknown. At least two ways of activating ERK have been reported that may not require G proteins, i.e. β-arrestin-mediated signaling (Jean-Charles et al., 2017) and transactivation of a receptor tyrosine kinases by a GPCR leading to ERK and Akt phosphorylation (Cattaneo et al., 2014). Based on our data, we cannot exclude that β-arrestin or receptor tyrosine kinases play a role in the activation of ERK and Akt. To study the role of non-classical routes to ERK activation, inhibitor studies, or probes that interrogate these processes, would be useful.

Increasing the number of probes to measure several processes simultaneously would provide a better picture of the contribution of different networks and their interactions. Multiplex, live-cell imaging with six probes has been demonstrated (Valm et al., 2017) and would enable the measurement of a reference for segmentation and five KTRs or other probes. Ongoing efforts to engineer brighter fluorescent proteins and hybrid genetic tags (e.g. HaloTags and SNAP tags) are important to further improve multiplex imaging. The functional translocation read-outs can potentially be combined with morphological profiling (Bray et al., 2016) for multiparameter, high-content imaging-based drug screens.

We hope that the new imaging strategy and analysis presented here will be valuable for future studies that use imaging of kinase activity in single cells to connect GPCR activation with physiological effects.

## MATERIALS AND METHODS

### Reagents

S1P (Sigma-Aldrich, S9666) was prepared as a 1.3 mM stock solution in methanol. Histamine (Sigma-Aldrich, H7125) was prepared as a 100 mM stock solution in water. UK 14304 (Sigma-Aldrich, U104) was prepared as a 10 µM stock solution in dimethyl sulfoxide (DMSO). YM-254890 (FUJIFILM Wako Pure Chemical Corporation, 257-00631) was prepared as a 1 µM solution in 33% DMSO in MQ water. PTx (Invitrogen, PHZ1174) was prepared as a 100 ng/ml solution in water. PD 0325901 (Sigma-Aldrich, PZ0162) was prepared as a 1 mM solution in DMSO.

### Cloning single KTRs and nuclear marker

The first step to generate the multicolor constructs was to clone the individual KTRs and the nuclear marker, and tag them with the fluorescent proteins of interest. The ERK-KTR, developed by Regot et al. (2014), and the Akt-KTR, by Maryu et al. (2016), were part of the pHGEA plasmid kindly shared by Dr Kazuhiro Aoki (National Institute for Basic Biology, Okazaki, Japan). Both sequences contained a P2A sequence in front, which we kept to ensure equimolar expression of the separate proteins from a single transcript (Kim et al., 2011).

P2A-ERK-KTR and P2A-Akt-KTR sequences were amplified by PCR from pHGEA (Akt: Fw 5′-TATAGGTACCAAACCATGGGGTCAGGGGCCACCAACTTC-3′ and Rv 5′-TATAACCGGTATGCGGCCGCCGAGCGTGATGTTATC-3′; ERK: Fw 5′-TATAGGTACCAAACCATGGGGAGCGGGGCTACCAACTTC-3′ and Rv 5′-ATATACCGGTATGCCGCCGGACGGGAATTG-3′) to introduce Acc65I and AgeI restriction sites (underlined in primer sequences). Next, the P2A-KTRs PCR products and Clontech N1 vectors containing either mTq2 (Goedhart et al., 2012), mNG (Shaner et al., 2013) or mScI (Bindels et al., 2016) were digested with Acc65I and AgeI, ligated using T4 DNA ligase, and transformed by heat-shock using DH5α *Escherichia coli* competent cells.

In addition, the residues S294 and S344 in the Akt-KTR were mutated to Ala (the mutation is underlined in the primer sequence) by site-directed mutagenesis (S294A: Fw 5′-CCAAGTGGCCTGGCGCCCCCACGTCACGCA-3′ and Rv 5′-TGCGTGACGTGGGGGCGCCAGGCCACTTGG-3′; S344A: Fw 5′-﻿TGCGCCTCTCGCGCCCATGCTCTACAGCAG-3′ and Rv 5′-﻿AGCATGGGCGCGAGAGGCGCATCATCGTCC-3′), as it has been reported that these residues in FOXO3 could be phosphorylated by ERK (Yang et al., 2008). We used PfuTurbo DNA polymerase, followed by DpnI digestion to destroy template DNA.

To generate the nuclear marker, we replaced mTq2 from a Clontech N1 H2A-mTq2 for mScI using AgeI and BsrGI.

### Combining KTRs

The second step was to combine the translocation reporters and the nuclear marker. Taking advantage of the compatible cohesive ends generated by digestion of Acc65I and BsrGI, we first generated P2A-Akt-KTR-mTq2-P2A-ERK-KTR-mNG by ligating P2A-ERK-KTR-mNG digested with Acc65I and P2A-Akt-KTR-mTq2 digested with Acc65I and BsrGI. Later, with the same approach, we generated H2A-mScI-P2A-Akt-KTR-mTq2-P2A-ERK-KTR-mNG, which we refer to as HSATEN. The plasmid is available from Addgene (plasmid #129631).

To incorporate HSATEN into the PiggyBac transposon vector pMP-PB (Matasci et al., 2011), kindly shared by Jakobus van Unen and David Hacker, we digested both constructs with NheI and XbaI and then ligated them. Because the cohesive ends generated by these enzymes are compatible, we performed a colony PCR to determine which colonies expressed the construct in the right orientation. We used transposon vectors containing antibiotic resistance for puromycin, blasticidin, hygromycin and zeocin. The plasmid with puromycin resistance was used in this study and is available from Addgene (plasmid #129632).

### Cell culture

HeLa cells (CCL-2, American Tissue Culture Collection, Manassas, VA) and HeLa stable cell lines were maintained in ‘full growth medium’, or Dulbecco's modified Eagle medium with GlutaMAX (Gibco, 61965059) supplemented with 10% FBS (Gibco, 10270106), at 37°C in 7% CO_2_ in humidifying conditions. Cells were passaged every 2–3 days by washing with HBSS (Gibco, 14170), trypsinizing using 0.25% Trypsin-EDTA (Gibco, 25200056), spinning down at 300 ***g*** for 5 min and resuspending in full growth medium. All cells were routinely tested for mycoplasma by PCR.

### Generation of HSATEN cell lines

HeLa cells (200,000) in 2 ml full growth medium were plated per well on a six-well plate (Corning, 3516) and left to grow overnight. The following day, we co-transfected 500 ng pPuro-PiggyBac-HSATEN and 200 ng transposase using 3.5 µl PEI (1 mg/ml in water). As a negative control, we transfected HSATEN and transposase. Twenty-four hours post-transfection, 1 μg/ml puromycin (Gibco, A1113803) was added to the cells, and, after 48 h, both the medium and puromycin were refreshed. After 72 h of selection with puromycin, the cells were trypsinized and passed to T25 flasks until confluency.

To sort by FACS, the cells were first washed, trypsinized, spun down and resuspended in full growth medium as for passaging. Then, the cells were spun down, resuspended in 2% FBS in HBSS containing 1 µg/ml 4′,6-diamidino-2-phenylindole (DAPI; Invitrogen, D1306), spun down, resuspended in DAPI-free 2% FBS-HBSS, and kept in the dark on ice. Cells were sorted with the FACSAria™ III (BD Biosciences, Franklin Lakes, NJ, USA), using a 100 µm nozzle at 20 psi pressure.

Single cells were identified by drawing gates using the area, width and height of forward scatter and side scatter, and living cells based on being DAPI negative. Living cells were identified based on the DAPI staining. To draw the gates for mNG- and mScI-positive cells, we used HeLa cells as a negative control. DAPI was excited with 405 nm and measured with a 450/50 nm bandpass emission filter. mNG and mScI were excited with 488 nm and 561 nm, respectively, and detected with 530/30 and 610/20 bandpass emission filters.

We selected four gates based on mNG intensity, distributed along the 50% brightest cells. We then sorted the pools into 15 ml tubes and single cells in 96-well plates. The tubes and plates contained full growth medium, supplemented with 10 mM HEPES and 1% penicillin/streptomycin (P/S) (Gibco, 15140148). Additionally, the 96-well plates were first coated with 14 µg/ml fibronectin in PBS for 1 h. The cells in the tubes were spun down, resuspended in full growth medium with 1% P/S and seeded in wells or flasks, depending on the number of cells. The medium of the 96-well plates was replaced the following day by full growth medium. The single clone populations were sequentially transferred to bigger wells/flasks to expand.

### Characterization of HSATEN cell lines

To test the dynamic range of the KTRs in the sorted cells, we used 5% FBS, given the strong stimulatory effect of the growth factors it contains on ERK and Akt activities. We found no correlation between expression level (inferred from fluorescence intensity) and response but observed that some of the brightest cells displayed lower responses. Therefore, we continued with cells from a pool with intermediate brightness.

For each of 13 monoclonal lines derived from this pool, we quantified the cellular fluorescence intensity prior to stimulation, and the translocation of the Akt-KTR and ERK-KTR in response to serum (Fig. S4). We selected five clones for further characterization and examined their response to high concentrations of histamine (100 µM), S1P (1.3 mM) and UK (10 nM). We decided to use clone E2 for further studies with these ligands due to higher brightness than the other clones.

### Live-cell imaging

For live-cell imaging, we used a TCS SP8 confocal microscope (Leica Microsystems, Wetzlar, Germany) equipped with a 10× air objective Plan Apo 0.40 NA and a Mercury lamp at 37°C. We excited mTq2, mNG and mScI with 440 nm DPSS, 488 nm Argon and 561 nm DPSS lasers. Fluorescence was detected using HyD detectors for mTq2 and mScI (452–500 nm and 590–675 nm) and a PMT detector for mNG (506–560 nm). The width of the detectors was controlled with sliders through Application Suite X (LAS X, Leica Microsystems).

The day before imaging, ∼120,000 cells were seeded in a glass-bottom eight-well µ-slide (Ibidi, 80827) in full growth medium. Two hours before imaging, the medium was removed and replaced with microscopy medium (MM) (20 mM HEPES pH 7.4, 137 mM NaCl, 5.4 mM KCl, 1.8 mM CaCl_2_, 0.8 mM MgCl_2_, 20 mM glucose) containing 0.033% DMSO or 1 µM YM-254890 (dissolved in 33% DMSO in water). PTx was added at the end of the day the cells were seeded, at a concentration of 100 ng/ml. We incubated the cells for 2 h with serum-free MM prior to imaging to reduce the basal kinase activities of ERK and Akt.

The acquired images had a 12-bit color depth and 1024×1024 pixels resolution. Images were acquired every 3.5 min, and each image was the average of four frames.

To keep the cells in focus, we executed Best Focus on the first well at the beginning of each time point, and the correction was extended to the rest of the wells. Ligands were pipetted to all wells during time point 7.

Ligand solutions were prepared in pre-warmed MM containing either DMSO or YM, depending on the experiment. Then, 100 μl of each ligand solution was added to the well containing 300 μl. For histamine and UK, the stock solutions were used to prepare the solution with the highest concentration, and serial dilutions were prepared from it. For S1P, the serial dilutions were prepared in methanol using gastight syringes (Hamilton, 1702 and 1710), and medium was added afterwards up to 100 µl. Ligand solutions were kept at 37°C for 10 min before pipetting to avoid cellular stress. For stimulation with 5% FBS, 20 µl pre-warmed FBS was added to each well containing 380 µl MM.

To determine the concentrations that yield minimum and maximum Akt/ERK activities for each ligand, we tested concentrations in the following ranges: 0.13–200 µM for histamine, 16–2600 nM for S1P and 0.13–10,000 pM for UK.

### Image processing

A reproducible image processing pipeline using Fiji, CellProfiler and R is available at https://github.com/JoachimGoedhart/Nuclear-translocation-analysis. The repository includes example data, code, a manual and the expected outcome as a graph. Below, we describe the steps in detail.

Processing of the raw images was performed using FIJI (Schindelin et al., 2012). The individual signals were not unmixed because the cross excitation and bleed through were close to zero. To facilitate segmentation of the nuclei, we first subtracted 250 counts from the mScI images to remove any counts in the cytoplasm due to overexpression of the H2A marker. To remove the background from the mTq2 channel images, we applied a rolling ball of 70 pixels radius and used these images for quantification of the mTq2 signals. For identification of the cell boundaries, we first applied a Gaussian blur with sigma 2 to smoothen the mNG images and then applied a manual threshold from 300 to 65,535 to obtain a binary mask.

### Segmentation and tracking of nuclei and cytoplasm

We used a custom-made CellProfiler (version 3.0.0) (McQuin et al., 2018) pipeline for segmentation, measurement of intensity and shape features, and tracking. We first used the processed mScI images to identify the primary objects, i.e. nuclei. We used a global threshold of 330 counts to separate pixels into background and foreground, and included objects with a diameter within 8–20 pixels. Clumped objects were identified and separated according to intensity. To identify the cells, we used the nuclear regions of interest (ROIs) as seeds in the binary mNG images. The nuclear ROIs were expanded up to 5 pixels in all directions as long as there was no background. The cytoplasmic ROIs were simply determined as a subtraction of the nuclear ROI from the cellular ROI. The nuclear and cellular ROIs were then tracked through the time lapses. These ROIs were identified as unique objects if the distance between their positions in consecutive images was lower or equal to 3 pixels. Finally, the size/shape features of the nuclear and cytoplasmic ROIs were exported, together with the intensity features of these ROIs in the processed mTq2 and raw mNG images.

### Data processing

We then used a custom-made R script to process the exported data from CellProfiler. First, we applied filters to exclude ROIs with mean intensity values lower than ∼260 counts and higher than ∼4000. In addition, we removed ROIs with an area lower than 100 pixels and average pixel radius of 1 or less. Then, we removed the objects that were not present in each time point as a single object. Finally, we calculated the C/N ratio per cell by dividing the mean intensities of both ROIs, for mTq2 and mNG channels.

Owing to the experimental setup, the imaging of the six wells is not simultaneous, as there is a delay of 0.5 min between each well and the subsequent one. To get C/N ratios at the exact same times and simplify later analysis, we applied a linear interpolation to the data. In addition, data were normalized by subtracting the average of two time points prior to stimulation (usually the 5th and 6th time point) from every data point.

AUC was defined as the sum of the C/N ratios from the time points 9–18, corresponding to 7–38.5 min post-stimulation.

### Concentration–response curves fitting

To estimate the EC50 for each condition, we fitted the data using a four-parameter logistic curve, with the function ‘drm’ from the package ‘drc’ (Ritz et al., 2015). For each concentration, we used the average of the average value per biological replicate. The response from the negative control was entered as a low concentration, as the log of 0 is undefined. The data and R script for fitting the data are available at https://github.com/JoachimGoedhart/GPCR-KTR.

### Trajectories clustering with R

To cluster the data, we decided to combine the data from the three ligands, in order to compare the heterogeneity of responses among the three ligands. In addition, we included data from three experiments in which only vehicle was added, to use as negative control. To speed up the analysis, we used a subset of 15,000 cells, equivalent to ∼20% of the total number of cells. We used two different clustering approaches, hierarchical clustering and k-means clustering, and applied these to the normalized ratios from the time points 9–18, corresponding to 7–38.5 min post stimulation.

For the hierarchical clustering, we first used the function ‘parDist’, from the package ‘parallelDist’ (https://cran.r-project.org/web/packages/parallelDist/index.html), to create a matrix with the calculated ‘distances’ between all the cells. These distances represent the (dis)similarity between any two trajectories, and we used two of the most commonly used distance metrics, Manhattan and Euclidean. We then used the function ‘hclust’ from the package ‘fastclust’ (https://cran.r-project.org/web/packages/fastcluster/index.html) to cluster the trajectories according to the values in the distance matrix, using the linkage methods Ward and Ward2. The result is a dendrogram that can be cut into a k number of clusters or at a certain ‘height’. The Ward method is commonly used with squared Euclidean distances, but it can be used with non-squared Euclidean distances (Szekely and Rizzo, 2005) or Manhattan distances (Strauss and Von Maltitz, 2017). The only difference between Ward and Ward2, is that Ward2 first squares all the given distances.

For k-means clustering, it is necessary to first indicate the number of clusters (k) to be used. The Euclidean distances are then calculated and used to cluster the cells into k clusters. We used the function ‘kmeans’ from the base R package ‘stats’.

### Cluster validation with R

Ideal clusters will be compact, well separated and connected. In other words, we want to minimize the intra-cluster variation, maximize the inter-cluster distances, and each object and its nearest neighbors to be in the same clusters (Handl et al., 2005). Compactness tends to increase with cluster size, whereas separation and connectedness decrease. There are many metrics that combine them and can be used to quantitatively compare different clustering methods and to determine the ideal number of clusters.

To validate our clustering results, we used six different metrics: BW ratio, Dunn index, average Silhouette width, Pearson correlation index, Calinski and Harabasz index (or variance ratio), and Connectivity. We define BW as the ratio between the average of all distances between elements of different clusters, and the weighted average (to cluster size) of averages of distances between elements within a cluster. The connectivity, using a neighborhood size of 25, was calculated using the function ‘connectivity’ from the package ‘clValid’ (Brock et al., 2008). The rest of metrics were calculated using the function ‘cluster.stats’ from the package ‘fpc’ (https://cran.r-project.org/web/packages/fpc/index.html).

### Data visualization

Data were visualized with R and the ggplot2 package, with PlotsOfData (Postma and Goedhart, 2019) or PlotTwist (Goedhart, 2020). The scripts to produce the figures in the main text are available at https://github.com/JoachimGoedhart/GPCR-KTR.

## Supplementary Material

Supplementary information

Reviewer comments
