# Supplementary material for: Single-cell imaging of ERK and Akt activation dynamics and heterogeneity induced by G-protein-coupled receptors
Source: J Cell Sci. 2022 Mar 11;135(6):jcs259685. doi: 10.1242/jcs.259685 (PMC8977056; doi:10.1242/jcs.259685)
Supplement: Supplementary information [file joces-135-259685-s1.pdf]

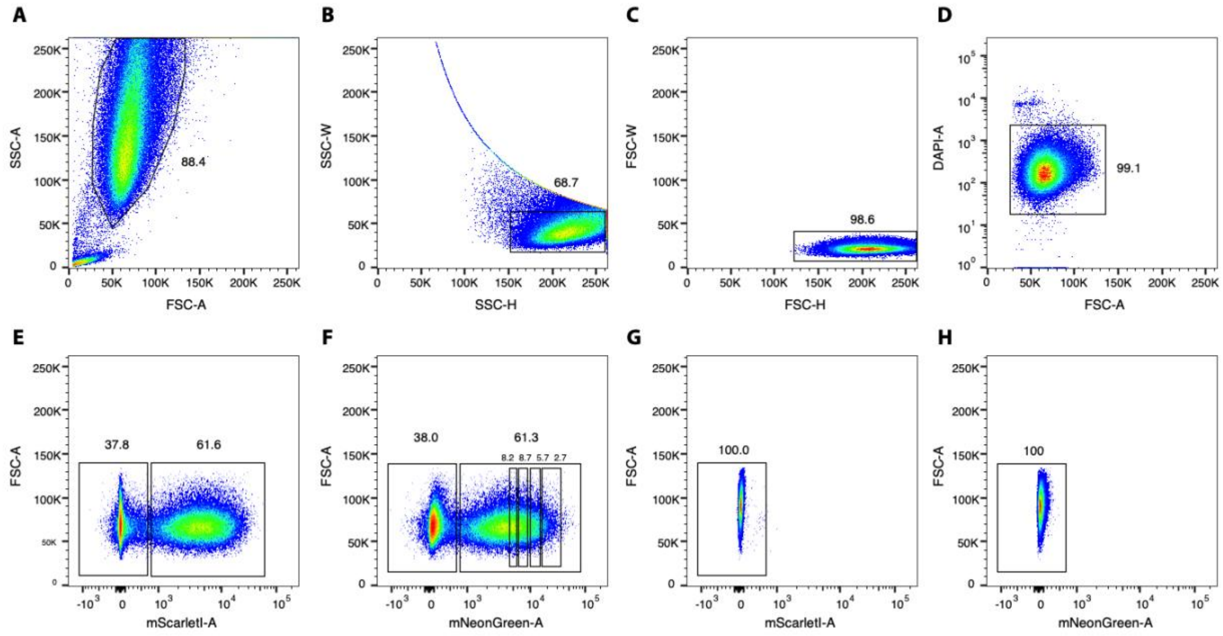

Fig. S1. FACS gating for HSATEN cells. A-F: HeLa cells stably transfected with PB-HSATEN post puromycin selection. A-C: Gating for single cells based on forward (FSC) and side scatter (SSC). D: Gating for living cells based on DAPI staining. E: Gating for mScI positive cells. F: Gating for mNeonGreen positive cells. G-H: HeLa controls cells. G: Gating for mScI negative cells. H: Gating for mNeonGreen negative cells.

Journal of Cell Science • Supplementary information

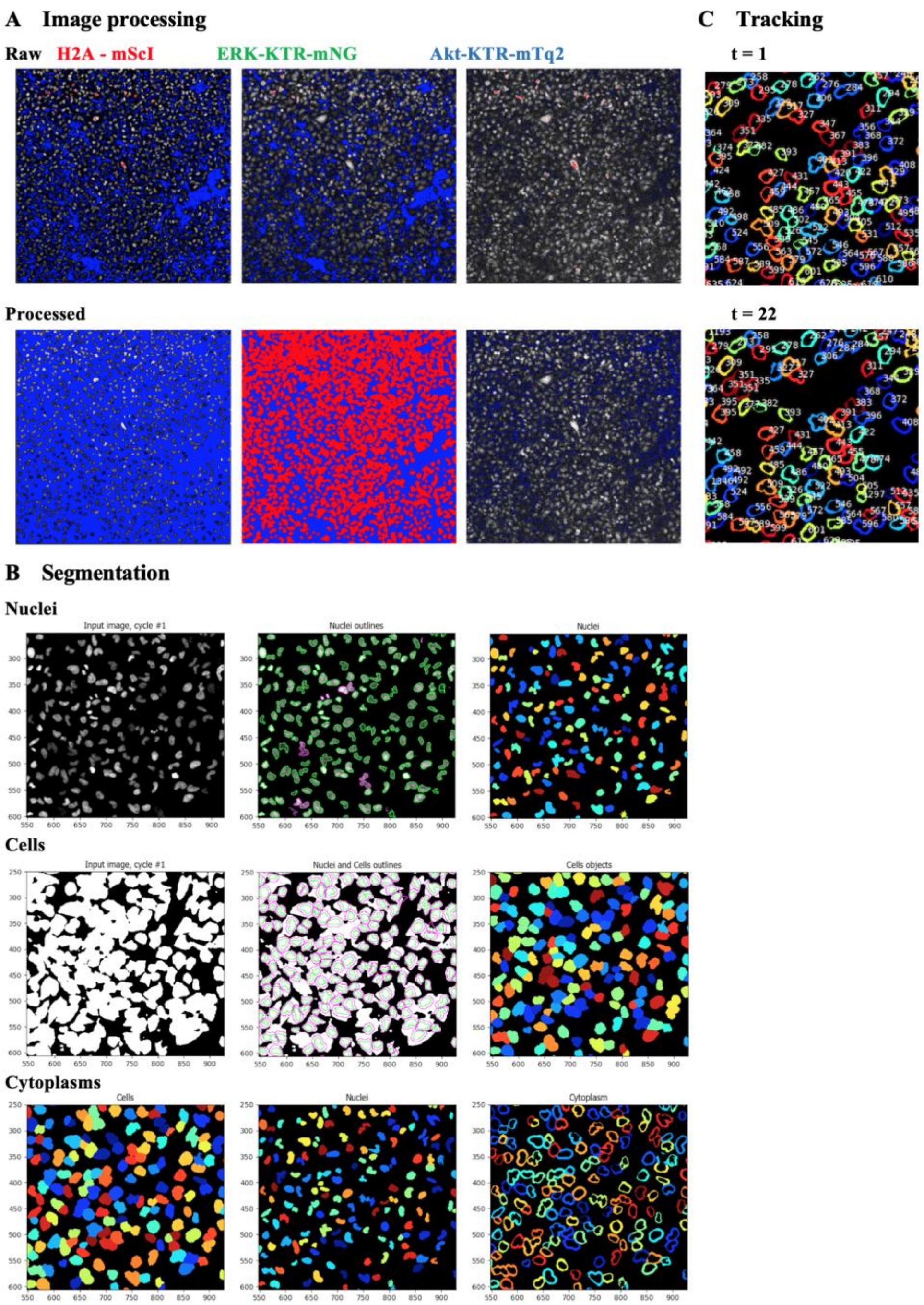

Fig. S2. Image processing, segmentation and tracking. A: Background subtraction and noise reduction of raw images with FIJI. Top: Raw images of the mScI, mNG and mTq2 channels from a monoclonal population of HeLa cells stably expressing HSATEN. Bottom: Top images after processing. For mScI, we subtracted 250 counts. For mNG, we applied a Gaussian blur with sigma 2 and a threshold from 300 to 65535 to create a binary mask. For mTq2, we used the 'Subtract Background' function in FIJI with a rolling ball of 70 pixels. All panels are visualized using a HiLo LUT, which displays the dimmest pixels as blue, the brightest pixels as red, and the rest as a grayscale. B: Segmentation of nuclei, cells, and cytoplasms in CellProfiler. The panels show zoomed areas of the entire field of view. For the nuclei: Left panel shows the nuclear input image, which is the processed mScI image from panel. Center panel shows segmented nuclear outlines, with accepted objects in green and discarded objects in purple. Right panel shows segmented nuclei. For the cells: Cellular segmentation uses the segmented nuclei as seeds. Left panel shows the input image, which is the binary mask from in panel. Center panel shows segmented cellular outlines, with nuclear objects in green and cellular areas in purple. Right panel shows segmented cells. For the cytoplasms: Cytoplasmic segmentation results of subtracting the nuclear areas from the cellular areas. Left and center panels show the segmented nuclei and cells. Right panel shows segmented cytoplasms. C: Tracking of cytoplasms with CellProfiler. The left panels show the tracking of cells in a single field of view at the first and last time points of a time lapse of 22 images. The right panels are zoomed regions. The nucleus and cytoplasm of a single cell is identified with a unique tracked object number, shown next to each cytoplasm outline.

Journal of Cell Science • Supplementary information

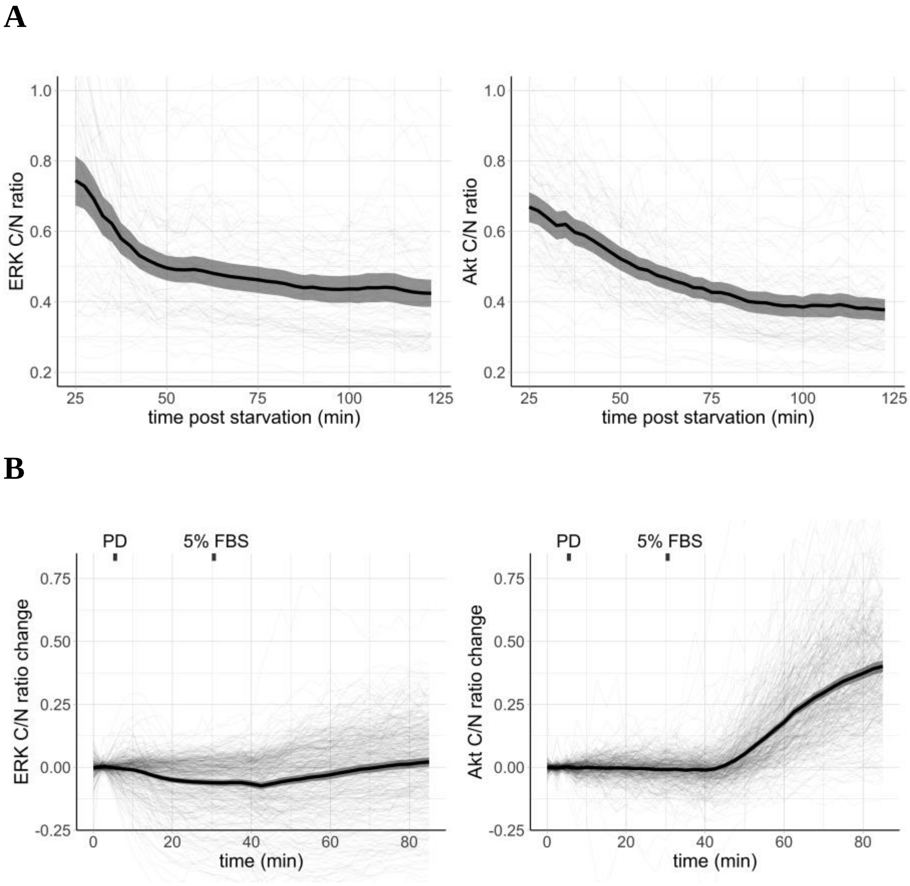

Fig. S3. Effect of serum starvation and MEK inhibition on ERK and Akt. A: Effect of serum starvation on the ERK and Akt C/N ratios. Cells were serum-starved at time 0 and the ratios were measured over time. Each panel shows combined data from at least three biological replicates. The line shows the average and the ribbon shows the standard deviation. B: Effect of MEK inhibition on serum-dependent activation of ERK and Akt. 1 µM PD 0325901 and 5% serum were added at the indicated time-points to serum-starved cells. Each panel shows a representative experiment from at least three biological replicates. The line shows the average and the ribbon shows the standard deviation.

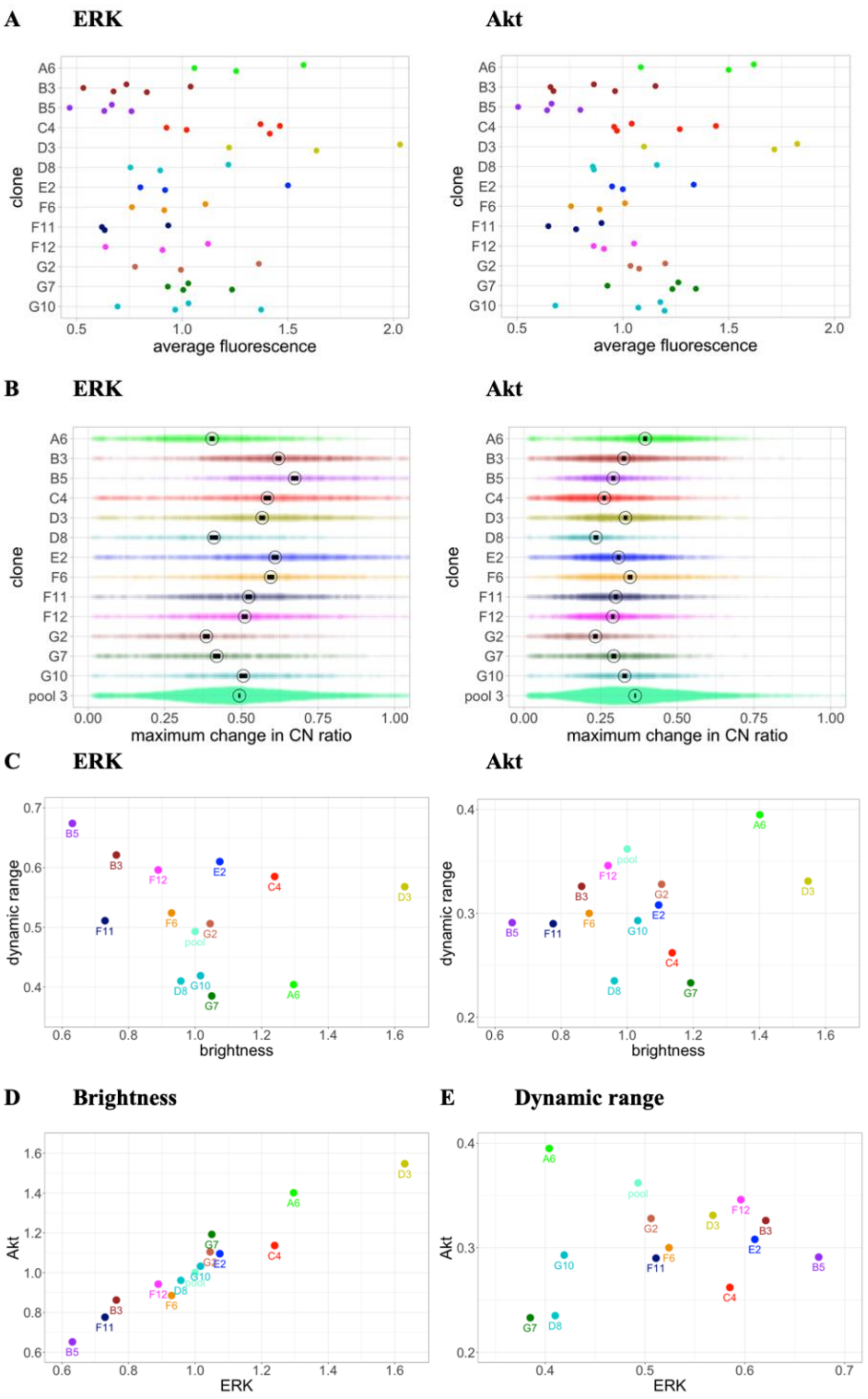

Fig. S4. Comparison of brightness and dynamic range of the 13 monoclonal populations originating from pool 3. A: Cellular fluorescence (arbitrary units) in the mTq2 (Akt-KTR) and mNG (ERK-KTR) channels. Each dot represents the average cellular fluorescence intensity in a biological replicate. For each cell and channel, the cellular fluorescence intensity was calculated as the average between time points 1 and 7, prior to stimulation with 5% FBS. B: Maximum change in C/N ratio for the Akt- and ERK-KTRs in response to 5% FBS. Each dot represents a single-cell value and corresponds to the highest C/N ratio after stimulation with 5% FBS. Each value is normalized by subtracting the average C/N ratio prior to stimulation. For each clone, the mark and the circle represent the mean and 95% CI of the mean. Plots were generated using PlotsOfData (Postma and Goedhart, 2019). C: The dynamic range of each clone plotted against the brightness for ERK and Akt respectively. D: Brightness of ERK and Akt per clone and E: the dynamic range of ERK-KTR versus that of Akt-KTR

Journal of Cell Science • Supplementary information

A

Histamine - ERK

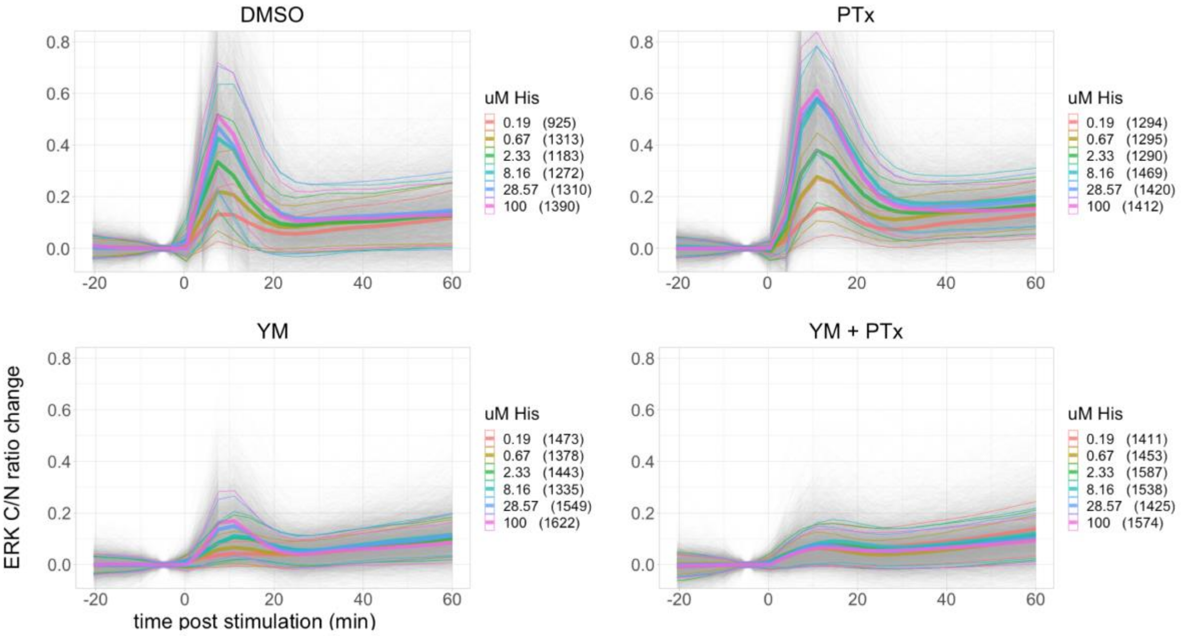

B

UK - ERK **UK - Akt**

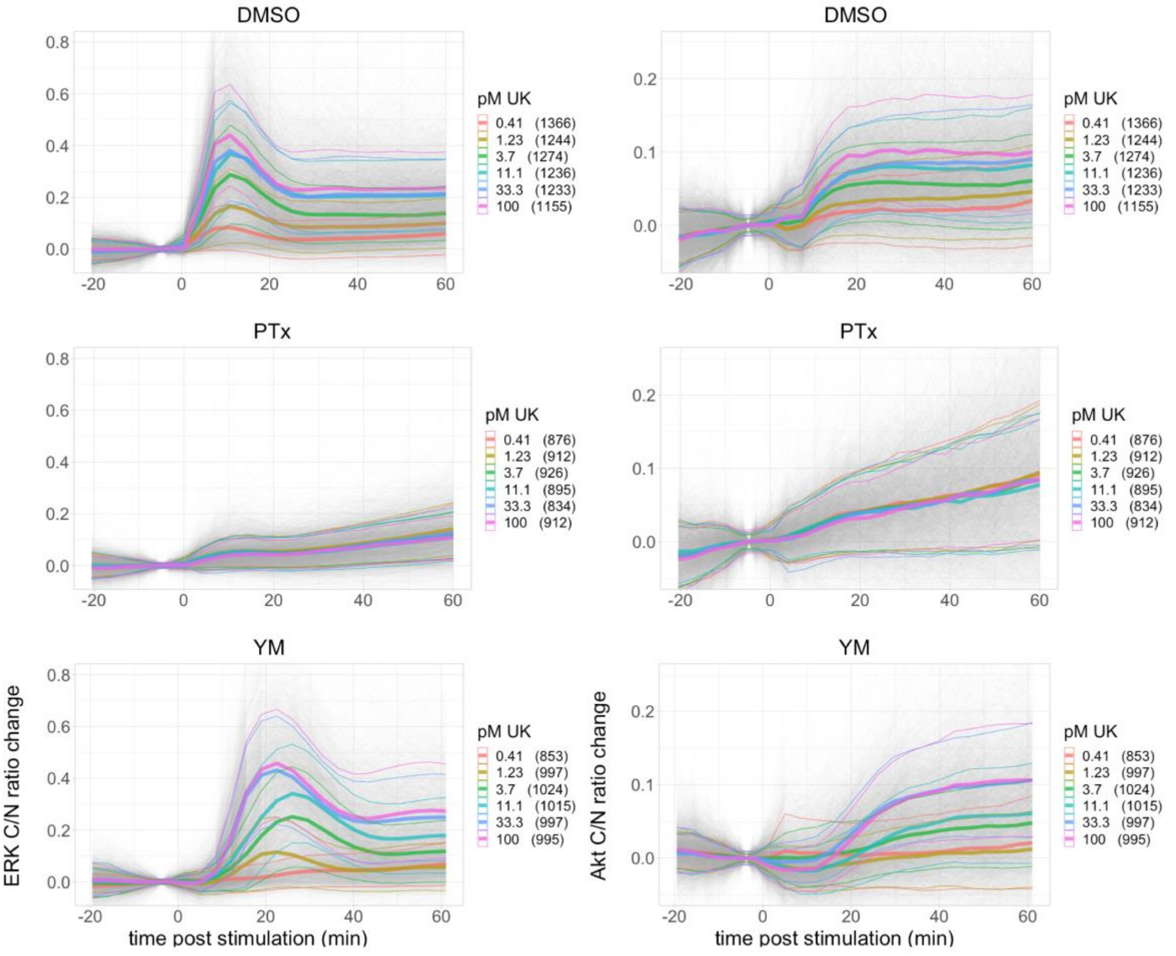

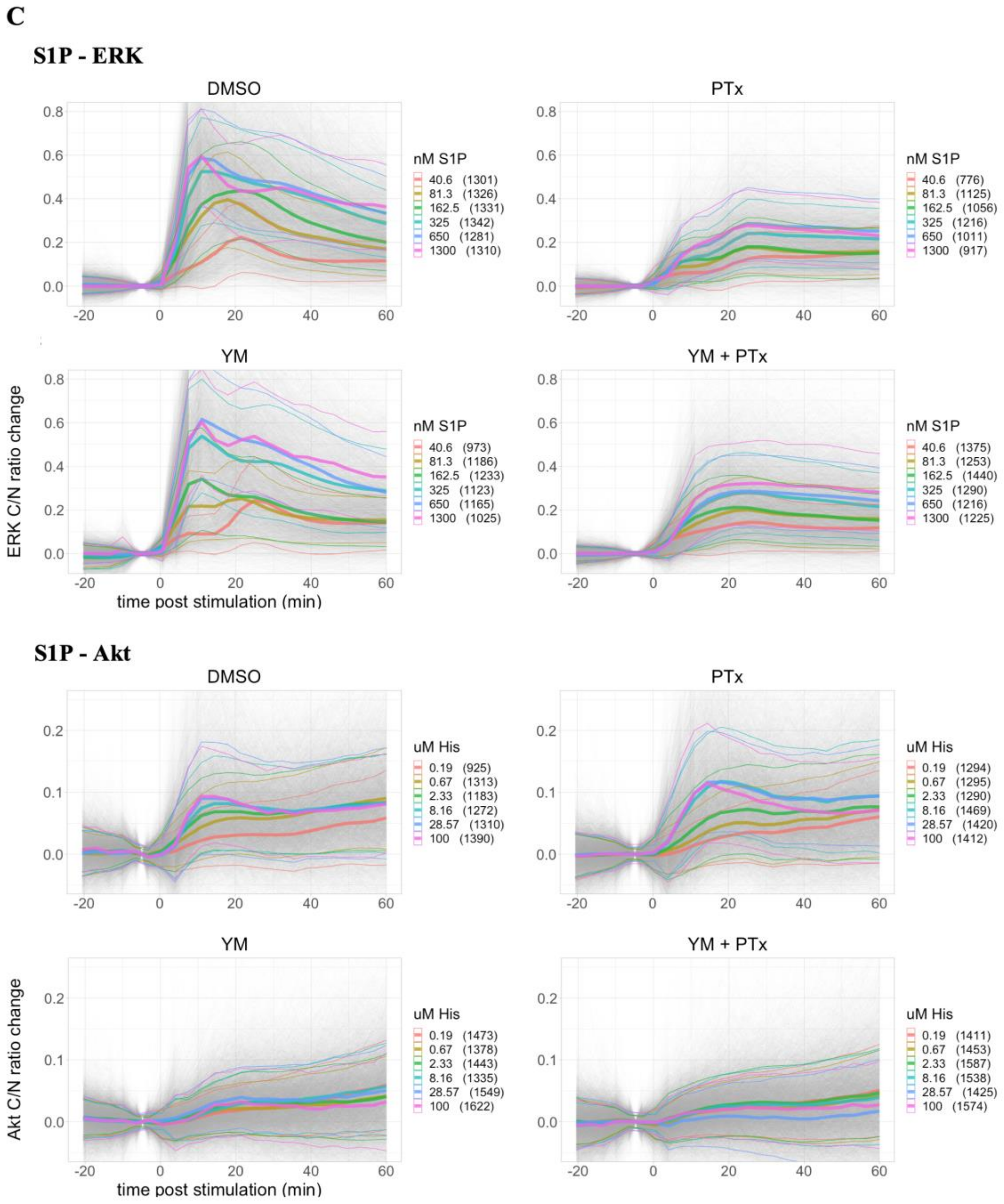

Fig. S5. ERK and Akt responses to histamine, UK, and S1P, and the effect of Gq and Gi inhibition. A: Histamine. B: UK. C: S1P. The different panels represent the following conditions: No inhibitor (DMSO), Gq inhibition (YM), Gi inhibition (PTx), and combined Gq and Gi inhibition (YM+PTx). C/N ratio change is calculated by subtracting the average from the two time points prior to stimulation. Each panel shows combined data from at least three biological replicates. Gray lines represent single cell traces. Thick colored lines show the mean and thin colored lines the standard deviation for each ligand concentration. Number of cells are shown between brackets.

Journal of Cell Science • Supplementary information

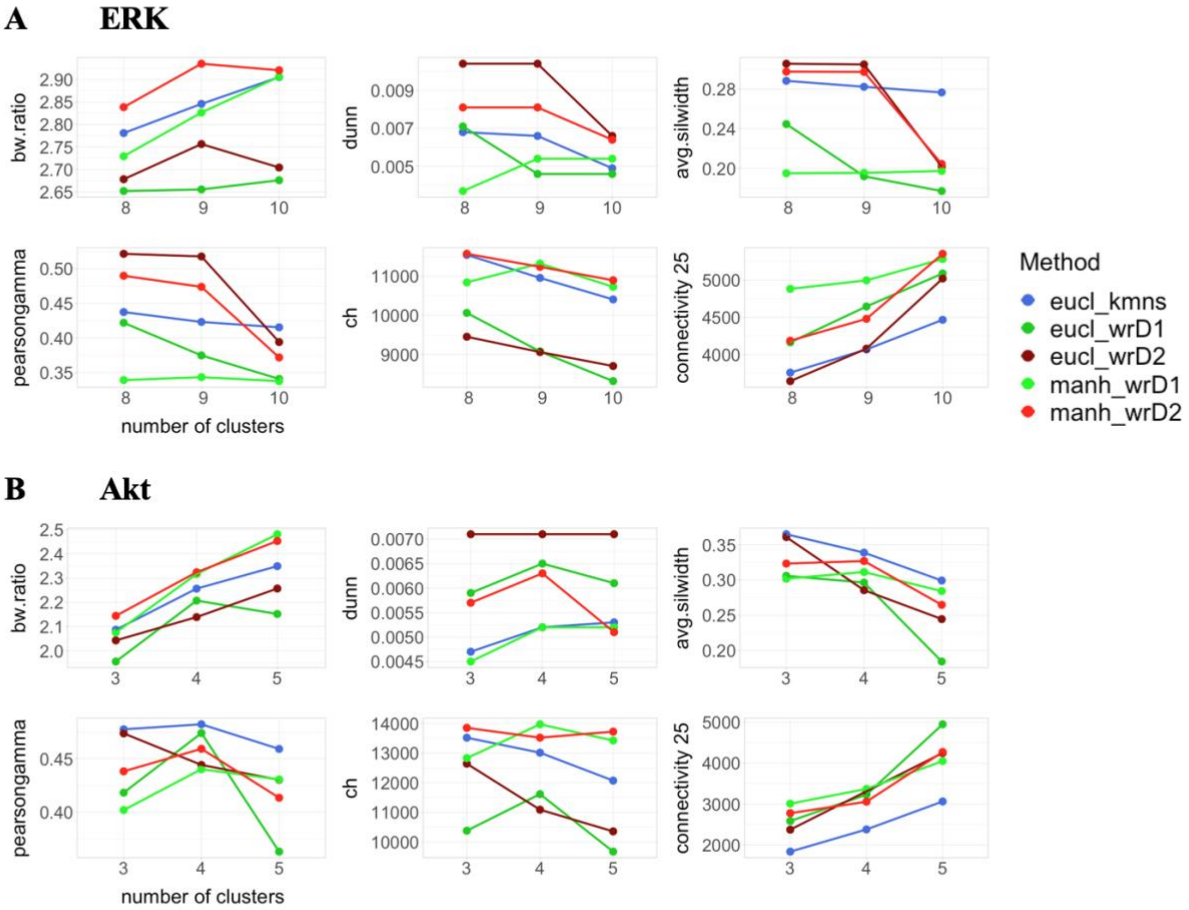

Fig. S6. Validation metrics scores for all tested clustering methods with various number of clusters. A: For ERK, 8 to 10 clusters. B: For Akt, 3 to 5 clusters. Each clustering method was applied to a subset of 15 000 cells from the combined experiments with different ligands, concentrations, conditions, and negative controls. Negative controls include cells preincubated with 0.03% DMSO, 1 µM YM, or 100ng/mL PTx where microscopy medium was added instead of ligand. The validation metrics include the BW ratio, Dunn index, average Silhouette width, Pearson correlation index, Calinski and Harabasz index, and Connectivity. In blue: k-means clustering. In dark green: Euclidean distance and Ward linkage method. In dark red: Euclidean distance and Ward2 linkage method. In green: Manhattan distance and Ward linkage method. In red: Manhattan distance and Ward2 linkage method.

Journal of Cell Science • Supplementary information

A ERK

Clusters distributions

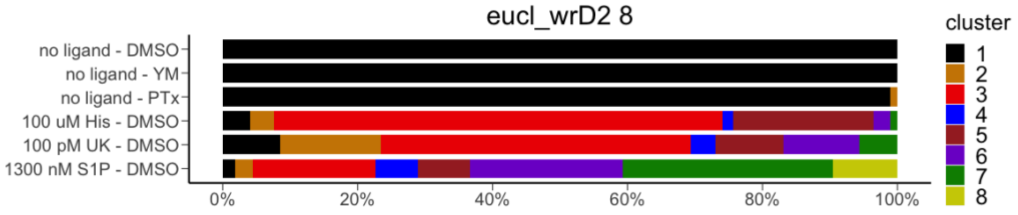

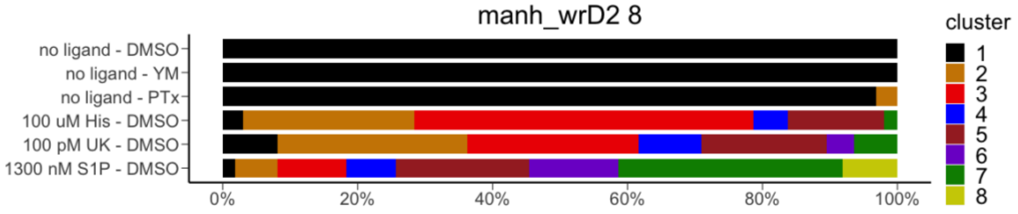

Clusters average response plots

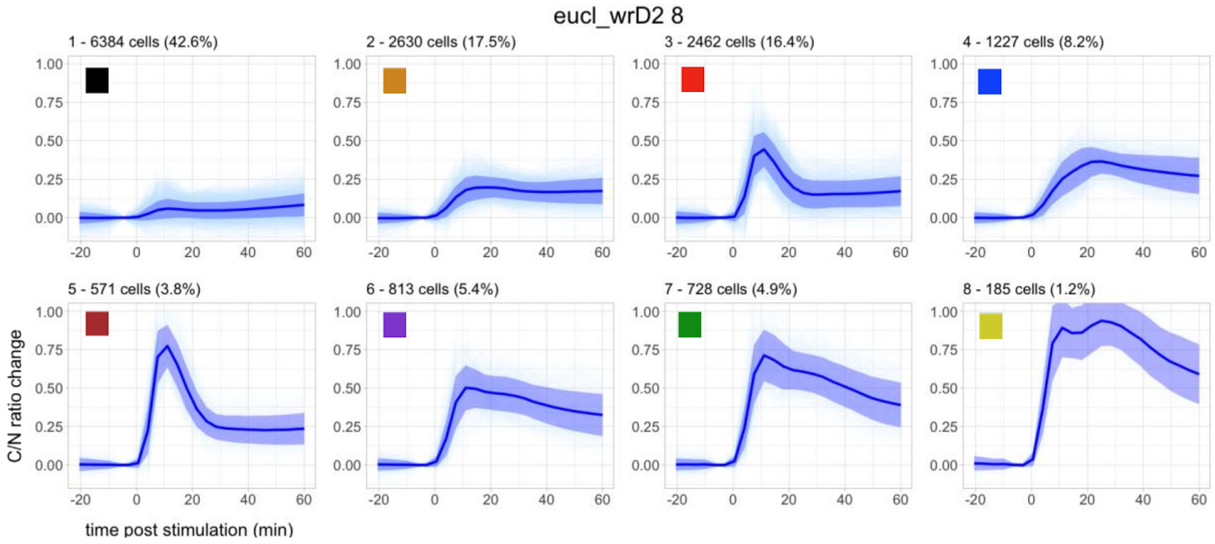

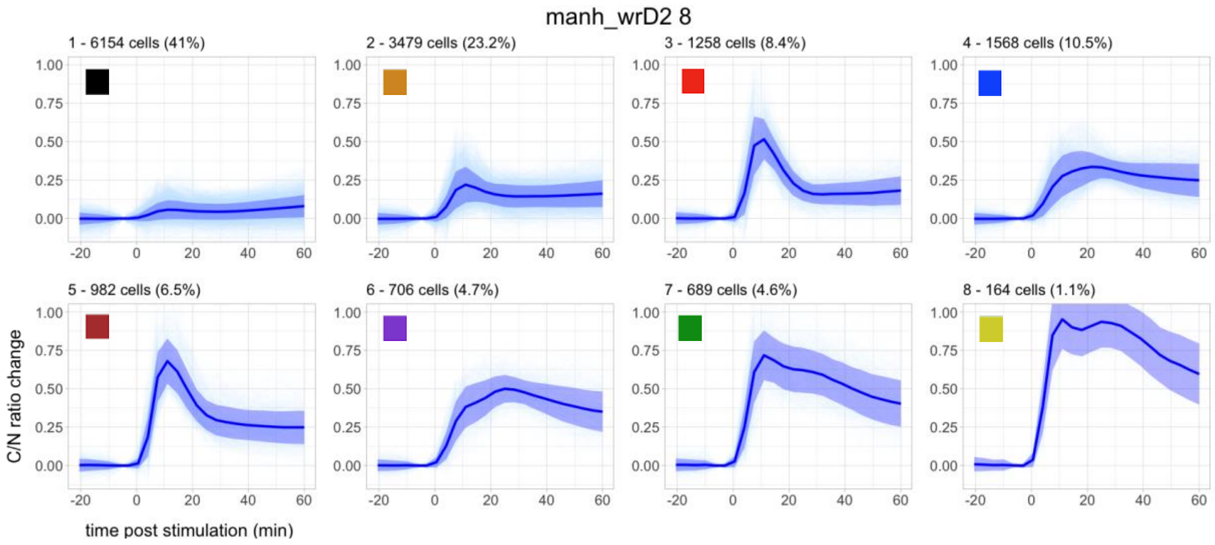

Journal of Cell Science • Supplementary information

B Akt

Clusters distributions

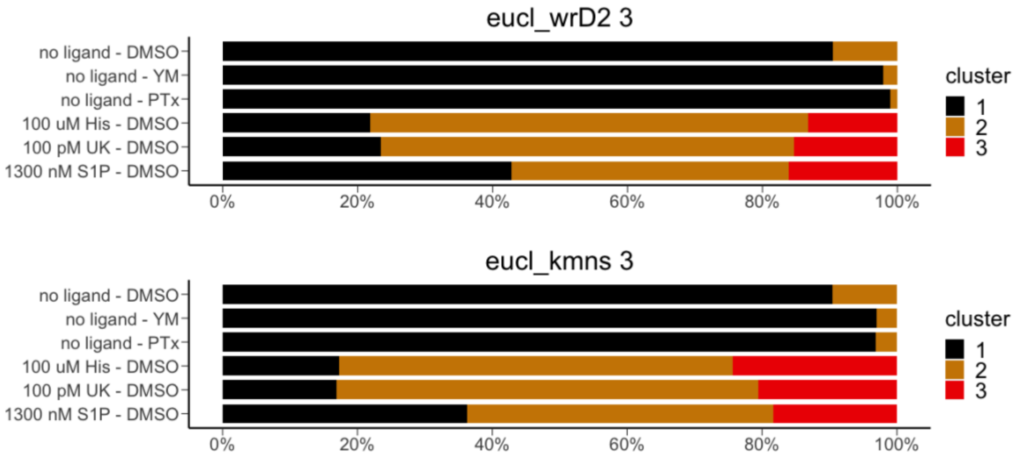

Clusters average response plots

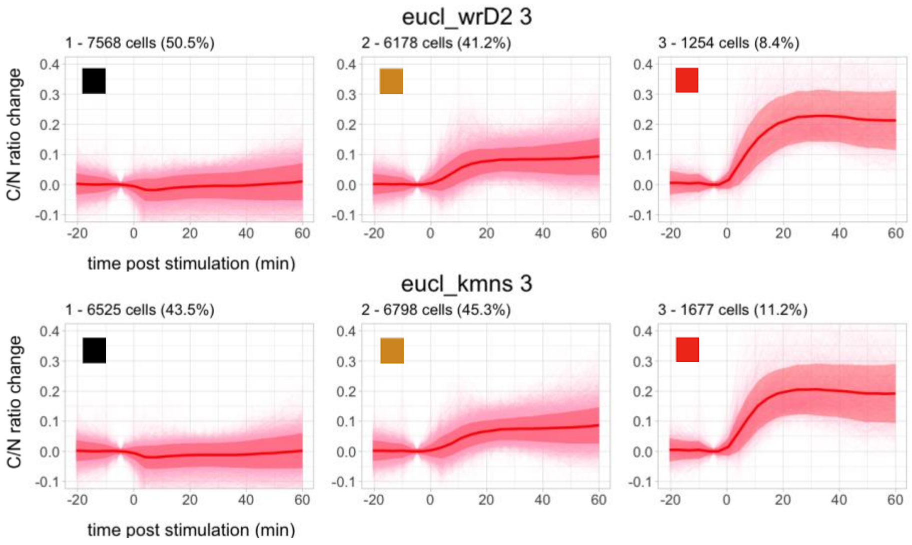

Fig. S7. Clustering candidates for ERK and Akt responses. A: ERK. The two selected methods have 8 clusters, use Ward2 linkage method, and use the Euclidean or Manhattan distance. B: Akt. The two selected methods have 3-4 clusters, use Ward2 linkage method, and use the Euclidean or Manhattan distance. Each method was applied to a subset of 15 000 cells from the combined experiments with different ligands, concentrations, conditions, and negative controls. First panel shows the cluster distribution of responses in negative and positive controls. Negative controls include cells preincubated with 0.03% DMSO, 1 µM YM, or 100ng/mL PTx where medium was added instead of ligand. Positive controls include cells preincubated with 0.03% DMSO where maximum stimulatory concentrations of Histamine, UK, and S1P were added. Second panel shows the average trajectory and frequency of each cluster. Per cluster, the lines represent the average trajectory and SD, and the number of cells and % of the total of 15 000 cells.

Journal of Cell Science • Supplementary information

A ERK

Histamine

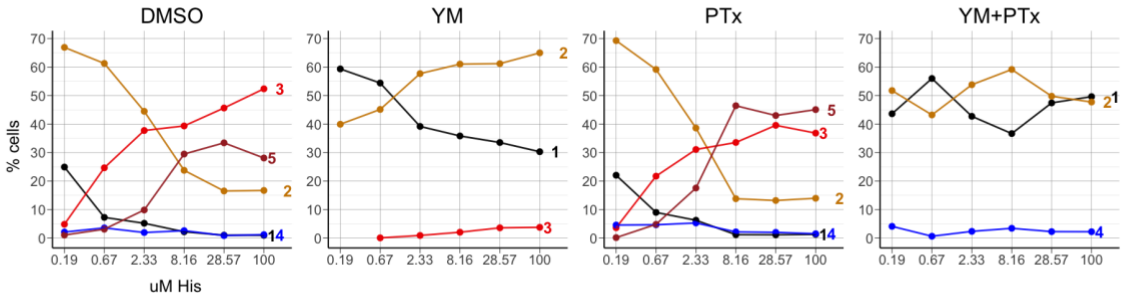

UK

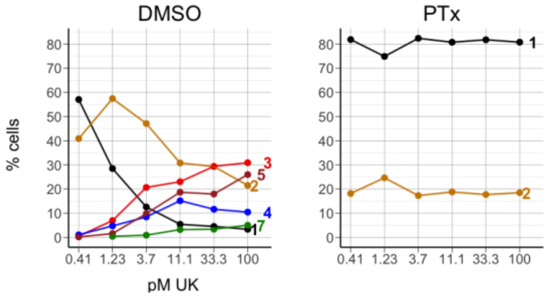

S1P

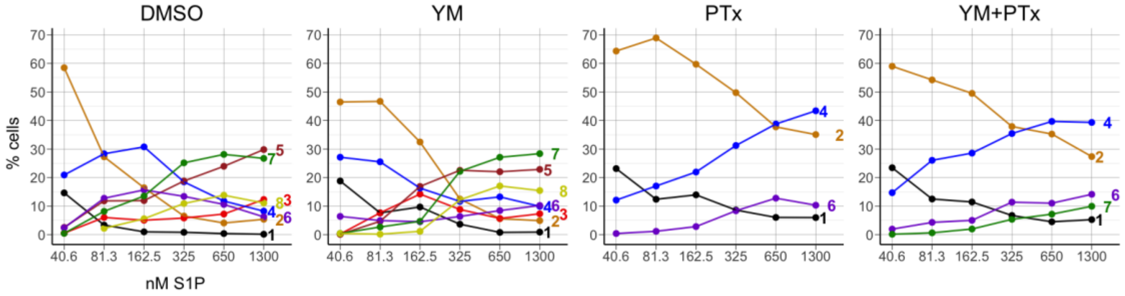

B Akt

Represented as bars

Histamine

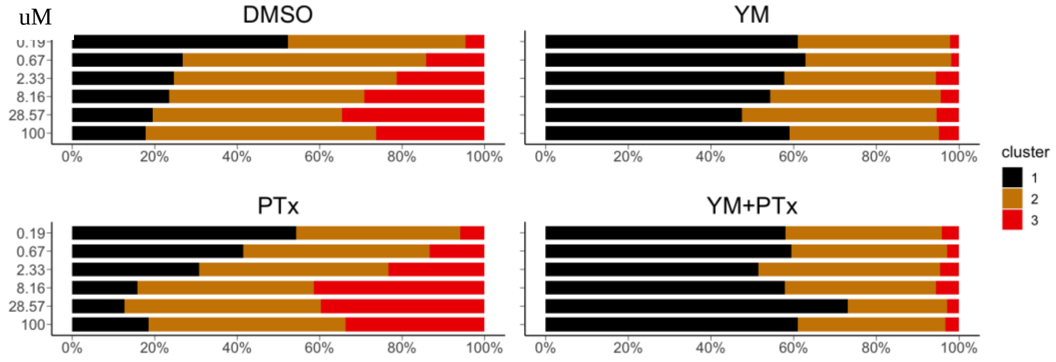

UK

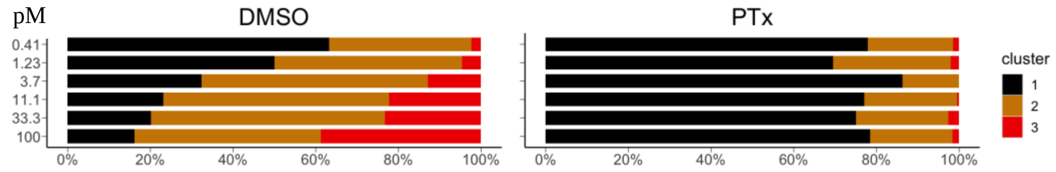

Journal of Cell Science • Supplementary information

S1P

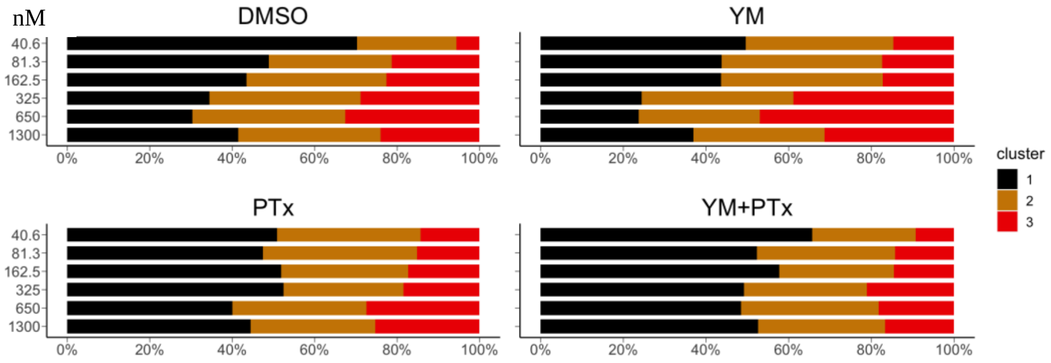

Represented as points

Histamine

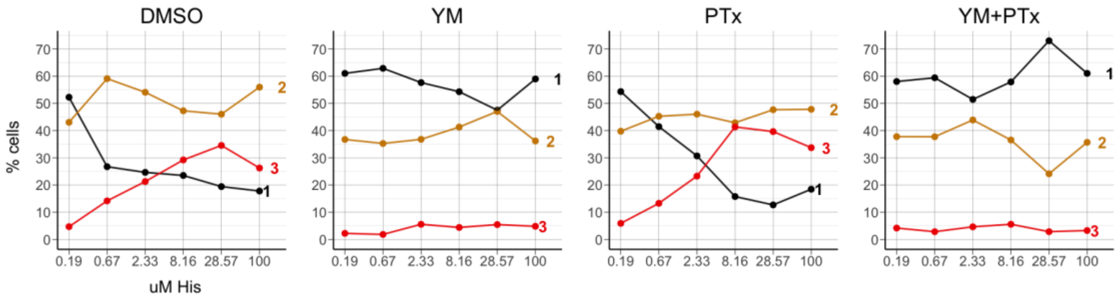

UK

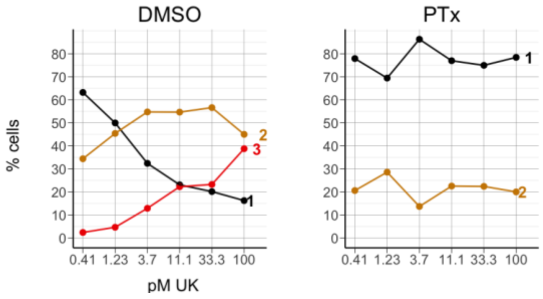

S1P

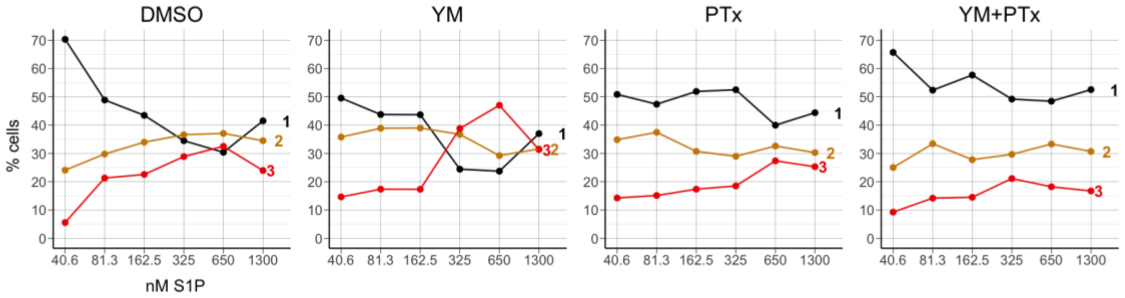

Fig. S8. Cluster distribution of ERK and Akt responses per ligand. A: Distribution of ERK responses shown as points. B: Distribution of Akt responses shown as bars and points. For each ligand, the panels represent the different experimental conditions: No inhibitor (DMSO), Gq inhibition (YM), Gi inhibition (PTx), and combined Gq and Gi inhibition (YM+PTx). For distribution represented as points, only clusters with an average frequency of 2% or more across all concentrations are shown.

Journal of Cell Science • Supplementary information

Table S1. Brightness and dynamic range of the 13 monoclonal populations originated from pool 3. The C/N change for Akt- and ERK-KTRs in response to 5% FBS is the average of the maximum C/N change of all the cells per clone across the biological replicates. Per cell, the maximum C/N change is the highest C/N ratio after stimulation with 5% FBS, normalized by subtracting the average C/N ratio prior to stimulation. Brightness in the mTq2 and mNG channels is expressed as the average of average cellular fluorescence from various biological replicates. For each replicate, the average was normalized to the average of two replicates of pool 3 in the same slide. For each cell and channel, the cellular fluorescence intensity was calculated as the average between time points 1 and 7, prior to stimulation with 5% FBS. n: number of biological replicates. In bold and blue, the 5 clones selected for further characterization.

		C/N ratio change		Brightness		
**Clone**	**# cells**	**Akt-KTR**	**ERK-KTR**	**mTq2**	**mNG**	**n**
A6	2327	0.4	0.4	1.4	1.3	3
**B3**	**2008**	**0.31**	**0.62**	**0.86**	**0.85**	**5**
B5	1267	0.28	0.67	0.65	0.63	4
C4	2054	0.24	0.6	1.14	1.42	5
**D3**	**2346**	**0.32**	**0.57**	**1.55**	**1.92**	**3**
D8	812	0.22	0.41	0.94	0.83	3
**E2**	**2138**	**0.3**	**0.62**	**0.91**	**0.84**	**3**
**F6**	**1679**	**0.32**	**0.59**	**0.89**	**0.93**	**3**
F11	1709	0.29	0.52	0.78	0.73	3
F12	1851	0.28	0.51	0.94	0.89	3
G2	1196	0.22	0.37	1.1	1.04	3
G7	1192	0.3	0.41	1.19	1.18	4
**G10**	**1150**	**0.33**	**0.5**	**1.03**	**1.14**	**4**
Pool 3	17466	0.35	0.48	1.0	1.0	16

Table S2. Parameters that describe the concentration-response curves fitted in Fig. 3 using ERK AUC as the measure of response. Half maximal effective concentration (EC50), Hill slope, and upper and lower limits.

Condition	Ligand	EC50	Hill	Lower	Upper
DMSO	His	0.28 µM	0.63	-0.60	2.97
PTx	His	0.41 µM	0.68	-0.43	3.30
YM	His	0.03 µM	0.52	-0.78	0.87
YM+PTx	His	0.03 µM	1.99	-0.33	0.66
DMSO	S1P	64 nM	1.49	-0.12	5.02
PTx	S1P	61 nM	0.66	-0.50	2.84
YM	S1P	140 nM	0.95	-0.16	6.02
YM+PTx	S1P	36 nM	0.48	-1.33	3.73
DMSO	UK	2.1 pM	0.89	-0.11	2.99
PTx	UK	0.34 pM	7.89	-0.03	0.24

Table S3. Sum of validation metrics for all the candidate clustering methods. Per kinase, the values from each of the six metrics were normalized by dividing them by the maximum score among the 15 combinations, and the sum of the six normalized metrics is shown. In bold and blue, the two highest scores per kinase.

	Akt		ERK	
**Method**	**k**	**Sum**	**k**	**Sum**
**eucl_kmns**	**3**	**5.46**	8	5.35
eucl_kmns	4	5.27	9	5.18
eucl_kmns	5	4.93	10	4.88
eucl_wrD1	3	4.78	8	4.94
eucl_wrD1	4	5.00	9	4.26
eucl_wrD1	5	4.05	10	4.02
**eucl_wrD2**	**3**	**5.47**	**8**	**5.73**
eucl_wrD2	4	4.91	9	5.61
eucl_wrD2	5	4.65	10	4.45
manh_wrD1	3	4.66	8	4.26
manh_wrD1	4	4.98	9	4.49
manh_wrD1	5	4.82	10	4.42
**manh_wrD2**	3	5.11	**8**	**5.53**
manh_wrD2	4	5.24	9	5.44
manh_wrD2	5	4.70	10	4.61
