# Supplementary material for: Single-cell imaging of ERK and Akt activation dynamics and heterogeneity induced by G-protein-coupled receptors
Source: J Cell Sci. 2022 Mar 11;135(6):jcs259685. doi: 10.1242/jcs.259685 (PMC8977056; doi:10.1242/jcs.259685)
Supplement: Reviewer comments [file joces-135-259685_review_history.pdf]

Single-cell imaging of ERK and Akt activation dynamics and heterogeneity induced by G-protein-coupled receptors

Sergei Chavez-Abiega, Max L.B. Grönloh, Theodorus W.J. Gadella Jr., Frank J. Bruggeman and Joachim Goedhart
DOI: 10.1242/jcs.259685

Editor: John Heath

Review timeline

Submission to Review Commons:	24 Aug 2021
Original submission:	15 December 2021
Editorial decision:	6 January 2022

Reviewer 1

Evidence, reproducibility and clarity

Summary

The authors have performed highly quantitative analyses of GPCR signaling to reveal heterogenous ERK and Akt activation patterns by using kinase translocation reporters. Using a massive number of single-cell imaging data, the authors show heterogeneous responses to GPCR agonists in the absence or presence of inhibitors. By cluster analysis, the responses of ERK and Akt were classified into eight and three patterns. This paper is clearly written with sufficient information for the reproducibility. However, the conclusion may not be necessarily supported by the provided data as described below.

Major comments:

This work has been well done in an organized way and adds new insight into the regulation of protein kinases by GPCRs. The conclusion will be of great interest in the field of single- cell signal dynamics and quantitative biology. On a bit negative note, considering the complexity of the downstream of GPCRs, some of the conclusions may need revision.
1. The conclusion of the title that "Heterogeneity and dynamics of ERK/Akt activation by GPCR depend on the activated heterotrimeric G proteins," may not be supported by the data. The authors compared just one pair each of GPCR and ligand. The heterogeneity may come from the nature of the ligand or the characteristics of the single clone chosen for this study.
2. The obvious question is that why the authors did not analyze the correlation between ERK and Akt activity more extensively. Cell Profiler will be able to extract multiple cellular features. Linking the heterogeneous signals to cellular features will benefit readers in the broad cell biology field. If the authors wish to write another paper with that data, it should be at least discussed.
3. Another apparent flaw of this work is that YM was not challenged to UK-stimulated cells. The authors probably assumed lack of effect. Nevertheless, I believe it is required to show. Or, remove the PTx data from the Histamine-stimulated cell data.
4. The most interesting response is that of S1P. ERK is biphasically activated. Combined inhibition of Gq and Gi failed to suppress ERK activity. It may be discussed why the biphasic activation pattern was not identified by the classification.
5. The authors argue that the brightness of the KTR reporter was not correlated with the dynamic range of ERK or Akt reporter (Supplementary Figure 3), but it is not clear. I had an impression that ERK-KTR brightness (Supplementary Figure 3A) has a slightly negative correlation with "maximum

© 2022. Published by The Company of Biologists under the terms of the Creative Commons Attribution License (https://creativecommons.org/licenses/by/4.0/).

change in CN ratio" (Supplementary Figure 3B) (e.g., A6>B3>B5 in brightness and A6<B3<B5 in maximum change in CN ratio). The authors should show dot plots of average fluorescence vs. the maximum change in CN ratio.
6. The authors have shown cluster analyses for the temporal patterns in kinase activations. However, the only difference of cluster 3 and 5 (Figure 7) seem to be amplitude. The authors have also shown the amplitude is dependent on the dose of the activators, which together makes it difficult to see the biological meaning of discriminating the two patterns in comparing different agonists, e.g., Histamine, UK, and S1P. The authors should discuss their views on how the clustering analyses will benefit biological interpretations together with possible limitations.
7. Considering the importance of the content, the supplemental note 2 may be included in the main text.

Minor comments:

1. The authors should clarify the cell type they used (HeLa cells) in the main text and figure legends.
2. Supplementary note1: The data-not-shown data (no correlation of KTR expression and its response to serum) should be very informative for the readers. The data should be shown as an independent supplementary figure.
3. Supplementary Figure S2: The authors should clarify this image processing is about background subtraction. Also, the authors should clearly note "rolling ball with a radius of 70 pixels" is about an ImageJ function, "Subtract Background".
4. Supplementary Figure S5: Figure labels are "A, A, B, B" not "A, B, C, D". Also the top two figures are lacking Y axis labels.
5. Page6 (top): The authors should mention the description is about Supplementary Figure S5 (UK) and Supplementary Figure S6 (S1P).
6. Figure 3: the figures are lacking x-axis labels (probably uM, nM and pM from left).
7. Values in tables: The significant figure must be 2, at best. This should be consistent throughout the text. For example, "The EC50 values for histamine, S1P and UK were respectively 0.3 ?M, 63.7 nM and 2.5 pM." This is somewhat awkward.
8. Page 7, the first paragraph: No comments on S1P!
9. Fig. 3: 100 mM must read as 100 micromolar.
10. Fig. 9: Concentration unit is missing.
11. Page 11, line 4: EKR should read as ERK.
12. Page 13: "So far, only a couple of studies looked into kinase activation by GPCRs and these studies used overexpressed receptors [32,33]." Please describe precisely. Protein kinase activation by GPCR has been studied more than 20 years. Why are these two recent papers cited here?
13. "This is in marked contrast to other fluorescent biosensors that typically require an overexpressed receptor for robust responses [34]." Following words should be included in the end: "in our hands".
14. "Histamine is reported to predominantly activate Gq in HeLa cells [36] and UK activates Gi [37]." Describe the name of receptors for the better understanding.
15. "S1P can activate a number of different GPCRs, all known to be expressed by HeLa cells [24]." Why is this paper chosen? The authors can easily find RNA-Seq data, if they wish to see the expression level. The cited paper did not scrutinize the S1P receptors expressed in HeLa cells.

Significance

The authors used biosensors for ERK and Akt to examine the kinetics of activation by GPCR ligands. Technical advancement is in the massive analysis method and cluster analysis. This is an important direction for the quantitative biology. GPCR signaling is complex because of multiple receptors coupled with different G proteins. The simple ones such as histamine receptor and alpha2-adrenergic receptor can be easily analyzed as shown in this study.
However, there are many S1P receptors, which make the interpretation difficult. If the authors could have shown interesting proposal on this data, the paper may interest many researchers in the field of cell biology and systems biology.

Expertise: Cell biology, signal transduction of protein kinases, fluorescence microscopy.

Referee Cross-commenting

© 2022. Published by The Company of Biologists under the terms of the Creative Commons Attribution License (https://creativecommons.org/licenses/by/4.0/).

1. I agree with the other two reviewers in that immunoblotting data is required to show the efficiency of P2A cleavage.
2. All reviewers think it looks strange that the authors did not show UK + YM data.
3. Showing the dynamic range of the biosensors will reinforce the data as Reviewer #3 states. ERK-KTR is quite sensitive and can be easily saturated. Ideally, the ratio of pERK vs ERK can be quantified by the different mobility in SDS-PAGE. But, I do not know how we can do it for Akt.

Reviewer 2

Evidence, reproducibility and clarity

Summary

In this paper Chavez-Abiega and colleagues investigate the dynamics of ERK and Akt activity downstream of several G protein-couples receptors (GPCRs). Using drugs to block specific G-proteins, they probe the activation of ERK/Akt by different heterotrimeric G proteins with fluorescent biosensors at the single cell resolution. Main finding is that ERK/AKT can be activated by different G-proteins, depending on the receptor coupling to the G-protein subclass, and that the ERK/AKT dynamics for S1P are specifically heterogeneous. Moreover, it seems that the AKT signaling response is very similar to ERK after GPCR stimulation.

Major points:

1) For this paper, the authors produced a new construct to express simultaneously the nuclear marker, the Akt and the ERK biosensors. The tree parts are connected by P2A peptides that determine their separation. Although, the biosensors are based on existing ones, the connection between them by P2A might create artifacts if the separation of the two parts is incomplete. For that, important controls are missing, such as treatment with an ERK and an Akt inhibitor. If the two parts are well separated the inhibitors should block the cytosol translocation of one of the two components and not of the other. This control is also important to check if in HeLa cells the Akt biosensors is not phosphorylated by ERK as well, as described in other reports. Alternatively, P2A separation can be quantified on a protein blot.
2) The description of ERK and Akt should be reported in a more uniform way, such as using the same representations for both (e.g. the equivalent of figure 2 for Akt is missing) or the same number of clusters.

3) Figure 3 & Figure 5: It seems that the YM and YM+PTx data for the UK 14304 data is missing. This would be an interesting addition to the manuscript, and it is easy to add. A similar analysis for the Akt sensor is missing in figure 3 and should be added for consistency. Figure 4 shows data for Akt, but as timeseries and only for Histamine. See point 2, it would benefit the reader greatly if ERK and AKT are presented in a more uniform and complete fashion throughout the manuscript.

4) In the results text of figure 4, the authors state that "...as shown in Figure 4C-D, which is in line with the effect of histamine on ERK.". It is unclear what the authors mean with this statement, the effects of single/double inhibition of Histamine stimulation on ERK are not quantified or discussed. Both responses can be quantified more carefully and compared.

5) This paper would benefit from a mechanistic investigation. For instance, the authors could investigate the pathways that lead to the generation of the pulse of ERK and Akt. These (preliminary) results presented call for deeper investigation into the signaling pathway from Gai and Gaq to ERK and AKT, and the authors are in a great position to probe this. One simple approach is to explore the upstream pathway, such as the MAPK cascade, PI3K, RTKs by means of inhibitors.

6) Since different G-proteins seem to elicit similar responses on ERK and especially for Akt, it is likely a B-arrestin / beta-gamma subunit mediated mechanism? It would be interesting to hear what the authors think of this, did they investigate/consider this possibility? E.g. Perhaps blocking RTK signaling / B-arrestin signaling would reduce heterogeneity?

© 2022. Published by The Company of Biologists under the terms of the Creative Commons Attribution License (https://creativecommons.org/licenses/by/4.0/).

7) The authors should take a serious effort to summarize the data in the figures better. Many plots that can be merged/presented in a more concise way, which would improve the readability of the manuscript greatly.

Minor points:

1) The authors should spell out in the legend of each figure if they are representing the absolute C/N or the normalized C/N

2) In Figure 2 the authors should show the control with no stimulus. Also would be informative to inform the reader about the stimulation protocol used, or indicate the stimulation time and length in the figure.

3) Figure 3: This figure would benefit from a different presentation of the data, it is currently confusing. E.g. Average curves per drug condition in a single graph would present the point the authors make more clear and concise, and this single cell overview can be moved to supplements.

4) Figure 4 legend states "CN ERK" and "ERK C/N", but is depicting only Akt responses? Only in 4c the axes are labeled, this together is very confusing.

5) Figure 5 is missing the controls with ERK and Akt inhibitors, to show the loss of correlation between the AUC of the two

6) Figure 6, the presumed lack of correlation between baseline activity and response should be confirmed statistically.

7) It seems that in S1P treated cells there is a second oscillation in ERK activity well visible in figure 2 and also in S10. Could the authors comment on that?

8) In the abstract it is unclear what authors mean with "UK".

9) Figure 9, it would be helpful to visually repeat the typical curve of the different clusters here, to guide the reader.

10) The observed heterogeneity in responses might be related to different cell cycle stages, did the authors investigated/consider this possibility (e.g. with a cell cycle biosensor)?

Significance

The paper describes with high accuracy the dynamics of ERK and Akt biosensors downstream of several GPCRs.

However, it feels like this is a preliminary report that leaves many important questions still open. It does not provide mechanistic insight and doesn't fully exploit the potential of single- cell technologies. The authors have the tools to investigate several important questions that are left open in the manuscript (e.g. connection Gaq/Gai to ERK/AKT, B-arrestin/betagamma involvement). Moreover, some important controls are missing. The authors should also consider the data presentation in the figures, to improve readability and interpretation of the manuscript.

Properly revised, would be of interest for a broad audience in cell biology, specifically GPCR and RTK signaling fields.

Expertise in cell biology, gpcr and rtk signaling, fluorescent biosensors.

Referee Cross-commenting

I agree with the assessments by the other reviewers.

Indeed showing the dynamic range of the biosensors, as Reviewer #3 states, would strengthen the manuscript and put the S1P response heterogeneity in context.

© 2022. Published by The Company of Biologists under the terms of the Creative Commons Attribution License (https://creativecommons.org/licenses/by/4.0/).

Reviewer 3

Evidence, reproducibility and clarity

This manuscript uses a live-cell biosensor approach to examine the activity kinetics of the ERK and Akt kinases in response to different GPCR ligands. The paper provides a detailed description of the development of a HeLa reporter cell line that expresses both Akt and ERK biosensors, along with a nuclear marker for use in cell tracking. The authors then catalog the individual responses from thousands of cells to three GPCR ligands. Individual cells show strong correlation in stimulated ERK and Akt activity. Using inhibitors for Gq and Gi proteins, it is shown that ERK and Akt activities are dependent on different G proteins. The authors also show that the heterogeneous responses within each population can be decomposed into several clusters representing similar dynamic behaviors; the frequencies of these clusters increase or decrease depending on treatments.
Overall, this is a well documented extension of an existing biosensor approach to examine GPCR signaling, and the approach is clearly described. There are however, some control experiments that are essential to support the conclusions.

Major comments:

1. The maximal responses of ERK and Akt biosensors in the selected cell clone are not adequately shown. Although FBS responsiveness is used as a validation and selection criterion, it would be much more informative to show the distribution of single-cell responses for defined activators of ERK and Akt, such as EGF and IGF-1, respectively. Without seeing the variability in these responses, it is difficult to put the heterogeneity observed in GPCR responses into context.
2. It is not clear whether the basal activity for the biosensors represents actual activity or simply the measurement floor. This should be established by using saturating treatment inhibitors for ERK and Akt to determine the biosensor readings in the absence of any activity. Ideally, an approach such as the one shown by Ponsioen et al. (PMID: 33795873) should be used to determine the dynamic range of the sensors.
3. Because the biosensors are separated by self-cleaving peptides, there is the potential that incomplete cleavage could complicate the results. Cleavage efficiency should be assessed by western blot or an equivalent method.
4. Ideally, an alternate method such as immunofluorescence for phosphorylated ERK/Akt or their substrates could be used in a subset of the conditions to validate the heterogeneity observed by the biosensors.

Minor comments:

1. In the introduction, more rationale and background could be provided for the examination of GPCR-stimulated ERK and Akt activity. There is not much information provided on why this is an interesting question. Other than the involvement of beta arrestin and RTK transactivation, which are mentioned, what mechanisms are known to be involved? Also, the importance of ERK and Akt in cancer is brought up, but it is not made clear how this approach or results would connect specifically to a cancer model.
2. It would be helpful to provide some explanation for why the UK+YM and UK+YM+PTx data are not shown in figure 3.
3. In the Abstract figure, it is not clear which samples "Inhibitor" and "Agonist" are referring to.

Significance

While similar reporter approaches have been used in a number of papers to examine growth factor signaling dynamics of ERK and Akt, this manuscript is the first I have seen to examine the responses of these kinases to different GPCR ligands. In doing so, it adds significantly to the growing body of literature on single-cell signaling responses. The mechanisms of ERK and Akt activation by GPCRs remain somewhat ambiguous, and the data reported here will be helpful in refining models for this signal transduction process. The findings that the GPCR ligands examined show different G protein dependencies than anticipated is an interesting facet, as is the observation that, while ERK and Akt are generally correlated, inhibition of Gi preferentially blocks S1P-induced ERK activity more so than Akt activity. However, the main findings of heterogeneity in signaling, and the observation of clusters that describe the different dynamic behaviors present within a population, are highly

© 2022. Published by The Company of Biologists under the terms of the Creative Commons Attribution License (https://creativecommons.org/licenses/by/4.0/).

consistent with what has been shown in other systems. Overall, this study is a useful confirmation that GPCR signaling to ERK and Akt follows a similar pattern to other forms of stimulation.

Referee Cross-commenting

Regarding the dynamic range, I don't think it is necessary to do a western blot (though this would be nice) - I think it would be sufficient to show maximal activation using EGF/IGF and full suppression using MEK/ERK and Akt inhibitors.
I also agree that all the points raised by the other reviewers. In particular, a deeper exploration and better visualization of the relationship between ERK and Akt would be very useful, as noted by both Reviewers #1 and #2.

Author response to reviewers' comments

1. General Statements [optional]

We were pleased with the generally positive evaluation of our preprint. All three reviewers see the value of the work: "*This work has been well done in an organized way and adds new insight into the regulation of protein kinases by GPCRs*" and "*The paper describes with high accuracy the dynamics of ERK and Akt biosensors downstream of several GPCRs*" and "*it adds significantly to the growing body of literature on single-cell signaling responses*". We agree with the reviewers that the molecular mechanisms that connect ERK and Akt with GPCR signaling are unclear and that many questions are still open. However, we'd like to stress that this is the first study using different GPCR ligands to look into kinase activity with these novel tools (as also appreciated by the reviewers). As such, it is a starting point for more detailed and mechanistic studies. In this manuscript we demonstrate the potential of this method and we develop tools and code to address new questions. The plasmids are available at addgene. For the analysis and visualization, we provide the scripts and use only open-source software, promoting the use of the tools that we developed.

2. Point-by-point description of the revisions

This section is mandatory. Please insert a point-by-point reply describing the revisions that were already carried out and included in the transferred manuscript.

First, we provide a brief overview of the main revisions that were done based on the Reviewers comments:
-We added data to figures 2,3,5
-We have revised figures 2,3,4,5,6,7,8,9 to include new analyses and to improve the data visualization.
-We have moved an explanation of how we choose the clustering methods to the main text
-We have rewritten all R-code, to improve re-usability, by (i) simplifying the code and (ii) adding comments. The code that generates the main figures is available on Github (https://github.com/JoachimGoedhart/GPCR-KTR/).
A point-by-point reply follows. Changes made to the text are stated in the answer and are indicated in blue in the revised document:

Reviewer #1 (Evidence, reproducibility and clarity (Required)):

Summary

The authors have performed highly quantitative analyses of GPCR signaling to reveal heterogenous ERK and Akt activation patterns by using kinase translocation reporters. Using a massive number of single-cell imaging data, the authors show heterogeneous responses to GPCR agonists in the absence or presence of inhibitors. By cluster analysis, the responses of ERK and Akt were classified into eight and three patterns. This paper is clearly written with sufficient information for the reproducibility. However, the conclusion may not be necessarily supported by the provided data as described below.

© 2022. Published by The Company of Biologists under the terms of the Creative Commons Attribution License (https://creativecommons.org/licenses/by/4.0/).

Major comments:

This work has been well done in an organized way and adds new insight into the regulation of protein kinases by GPCRs. The conclusion will be of great interest in the field of single-cell signal dynamics and quantitative biology. On a bit negative note, considering the complexity of the downstream of GPCRs, some of the conclusions may need revision.

We thank the reviewer for the evaluation and for raising a number of comments that have helped us to strengthen the manuscript and that will be addressed below.

1. The conclusion of the title that "Heterogeneity and dynamics of ERK/Akt activation by GPCR depend on the activated heterotrimeric G proteins," may not be supported by the data. The authors compared just one pair each of GPCR and ligand. The heterogeneity may come from the nature of the ligand or the characteristics of the single clone chosen for this study.

The title may suggest that the heterogeneity depends only on the G-protein (although that is not what the title says). Instead, we mean that G-proteins play a role in the heterogeneity, as we infer from the experiments with the G-protein inhibitors. If the reviewer feels strongly about this, we are open to changing the title, for instance to:
"Kinase translocation reporters reveal the single cell heterogeneity and dynamics of ERK and Akt activation by G protein-coupled receptors"

2. The obvious question is that why the authors did not analyze the correlation between ERK and Akt activity more extensively. Cell Profiler will be able to extract multiple cellular features. Linking the heterogeneous signals to cellular features will benefit readers in the broad cell biology field. If the authors wish to write another paper with that data, it should be at least discussed.

We agree that we can add more information on the correlation between ERK and Akt activity and we have added a plot that shows the co-incidence of the ERK and Akt clusters. This is now panel C of figure 8. We have no wish of writing another paper and we have made the data and code available, so anyone can do a more detailed analysis if desired.
We appreciate the suggestion to correlate activities with cellular features, such as cell area and shape. However, in our analysis we use nuclear fluorescence to segment the nuclear and cytoplasmic fluorescence (as generally done in studies that use KTRs). Therefore, the information on cellular features is not readily available. Such analysis would require a marker for the cytoplasm or membrane (or yet another image analysis procedure).

3. Another apparent flaw of this work is that YM was not challenged to UK-stimulated cells. The authors probably assumed lack of effect. Nevertheless, I believe it is required to show. Or, remove the PTx data from the Histamine-stimulated cell data.

We agree that this is valuable data to include. Unfortunately, this experiment was done in a slightly different condition than the other experiments (different spacing of the time intervals) and we initially skipped the data for these reasons. After careful examination of the data, we have decided to include these data (added to figures 3 & 5).
We note that we still miss the data from the YM+PTx data for UK and we have currently no way to carry out these experiments (mainly due to lack of funding). In our opinion, the absence of this data is not critical for the interpretation of the results. We prefer to show the YM+PTx data for the other two conditions.

4. The most interesting response is that of S1P. ERK is biphasically activated. Combined inhibition of Gq and Gi failed to suppress ERK activity. It may be discussed why the biphasic activation pattern was not identified by the classification.

We think that the biphasic activation pattern is reflected by cluster 7 and 8 and we now mention this in the text: "The biphasic ERK activation pattern, which is specific for stimulation with S1P are reflected by cluster 7 and 8."
For clarity, we now added the dynamics for each cluster to figure 9.

© 2022. Published by The Company of Biologists under the terms of the Creative Commons Attribution License (https://creativecommons.org/licenses/by/4.0/).

5. The authors argue that the brightness of the KTR reporter was not correlated with the dynamic range of ERK or Akt reporter (Supplementary Figure 3), but it is not clear. I had an impression that ERK-KTR brightness (Supplementary Figure 3A) has a slightly negative correlation with "maximum change in CN ratio" (Supplementary Figure 3B) (e.g., A6>B3>B5 in brightness and A6<B3<B5 in maximum change in CN ratio). The authors should show dot plots of average fluorescence vs. the maximum change in CN ratio.

We thank the reviewer for the suggestion and have now added this data to supplemental figure 3 as panel C.

6. The authors have shown cluster analyses for the temporal patterns in kinase activations. However, the only difference of cluster 3 and 5 (Figure 7) seem to be amplitude. The authors have also shown the amplitude is dependent on the dose of the activators, which together makes it difficult to see the biological meaning of discriminating the two patterns in comparing different agonists, e.g., Histamine, UK, and S1P. The authors should discuss their views on how the clustering analyses will benefit biological interpretations together with possible limitations.

This is a valid point, and it is a consequence of clustering method. We have added text to the discussion to explain our view: “The clustering is a powerful method for the detection of patterns and simplification of large amounts of data. Yet, it should be realized that clustering is mathematical procedure that is not necessarily reflecting the biological processes. One example is the graded response of ERK and Akt activities to ligands, whereas cells are grouped in weak, middle and strong responders. This may be solved by developing and using clustering methods that take the underlying biological processes into account.”

7. Considering the importance of the content, the supplemental note 2 may be included in the main text.

We appreciate this suggestion, and we have incorporated supplemental note 2 in the main text.

Minor comments:

1. The authors should clarify the cell type they used (HeLa cells) in the main text and figure legends.

This information is now indicated in the first paragraph of the results section and in the legend of figure1.

2. Supplementary note1: The data-not-shown data (no correlation of KTR expression and its response to serum) should be very informative for the readers. The data should be shown as an independent supplementary figure.

This relates to major point 5 and we agree that this is valuable. The data of the expression and the maximum response has been added to supplementary figure 3 as panel C.

3. Supplementary Figure S2: The authors should clarify this image processing is about background subtraction. Also, the authors should clearly note "rolling ball with a radius of 70 pixels" is about an ImageJ function, "Subtract Background".

We added text to highlight that the processing is a background subtraction and noise reduction. We added text to explain it is a FIJI function.

4. Supplementary Figure S5: Figure labels are "A, A, B, B" not "A, B, C, D". Also the top two figures are lacking Y axis labels.

Thanks for pointing this out. We the labels are corrected.

5. Page6 (top): The authors should mention the description is about Supplementary Figure S5 (UK) and Supplementary Figure S6 (S1P).

© 2022. Published by The Company of Biologists under the terms of the Creative Commons Attribution License (https://creativecommons.org/licenses/by/4.0/).

This is an accidental omission, it is corrected.

6. Figure 3: the figures are lacking x-axis labels (probably uM, nM and pM from left).

Well spotted, this is fixed by adding the units to the labels for each ligand.

7. Values in tables: The significant figure must be 2, at best. This should be consistent throughout the text. For example, "The EC50 values for histamine, S1P and UK were respectively 0.3 µM, 63.7 nM and 2.5 pM." This is somewhat awkward.

This has been fixed in the text and in the table.

8. Page 7, the first paragraph: No comments on S1P!

We added our observation that: “The response to S1P is hardly affected by YM, but the amplitude is reduced by PTx.”

9. Fig. 3: 100 mM must read as 100 micromolar.

We do not understand this comment, but the units of figure 3 are now corrected (see also point 6).

10. Fig. 9: Concentration unit is missing.

Thanks for pointing this out, units are added.

11. Page 11, line 4: EKR should read as ERK.

Fixed

12. Page 13: "So far, only a couple of studies looked into kinase activation by GPCRs and these studies used overexpressed receptors [32,33]." Please describe precisely. Protein kinase activation by GPCR has been studied more than 20 years. Why are these two recent papers cited here?

We updated the text to explain that: “So far, only a couple of studies looked into kinase activation by GPCRs in single cells with KTRs and these studies used overexpressed receptors”.

13. "This is in marked contrast to other fluorescent biosensors that typically require an overexpressed receptor for robust responses [34]." Following words should be included in the end: "in our hands".

We’ve included the suggested line.

14. "Histamine is reported to predominantly activate Gq in HeLa cells [36] and UK activates Gi [37]." Describe the name of receptors for the better understanding.

We added names: “Histamine is reported to predominantly activate Gq in HeLa cells by the histamine H1 receptor [36] and UK activates Gi by α2-adrenergic receptors [37]”

15. "S1P can activate a number of different GPCRs, all known to be expressed by HeLa cells [24]." Why is this paper chosen? The authors can easily find RNA-Seq data, if they wish to see the expression level. The cited paper did not scrutinize the S1P receptors expressed in HeLa cells.

The S1PR levels are scrutinized in the cited paper, but it is ‘hidden’ in the supplemental figure S4A. We will clarify this and explicitly mention this supplemental figure: “The situation for S1P is different. S1P can activate a number of different GPCRs, all known to be expressed by HeLa cells as shown in the supplemental figure S4A of [24]”

© 2022. Published by The Company of Biologists under the terms of the Creative Commons Attribution License (https://creativecommons.org/licenses/by/4.0/).

Reviewer #1 (Significance (Required)):

The authors used biosensors for ERK and Akt to examine the kinetics of activation by GPCR ligands. Technical advancement is in the massive analysis method and cluster analysis. This is an important direction for the quantitative biology. GPCR signaling is complex because of multiple receptors coupled with different G proteins. The simple ones such as histamine receptor and alpha2-adrenergic receptor can be easily analyzed as shown in this study. However, there are many S1P receptors, which make the interpretation difficult. If the authors could have shown interesting proposal on this data, the paper may interest many researchers in the field of cell biology and systems biology.

Expertise: Cell biology, signal transduction of protein kinases, fluorescence microscopy.

Referee Cross-commenting

1. I agree with the other two reviewers in that immunoblotting data is required to show the efficiency of P2A cleavage.
2. All reviewers think it looks strange that the authors did not show UK + YM data.
3. Showing the dynamic range of the biosensors will reinforce the data as Reviewer #3 states. ERK-KTR is quite sensitive and can be easily saturated. Ideally, the ratio of pERK vs ERK can be quantified by the different mobility in SDS-PAGE. But, I do not know how we can do it for Akt.

Reviewer #2 (Evidence, reproducibility and clarity (Required)):

Summary

In this paper Chavez-Abiega and colleagues investigate the dynamics of ERK and Akt activity downstream of several G protein-couples receptors (GPCRs). Using drugs to block specific G-proteins, they probe the activation of ERK/Akt by different heterotrimeric G proteins with fluorescent biosensors at the single cell resolution. Main finding is that ERK/AKT can be activated by different G-proteins, depending on the receptor coupling to the G-protein subclass, and that the ERK/AKT dynamics for S1P are specifically heterogeneous. Moreover, it seems that the AKT signaling response is very similar to ERK after GPCR stimulation.

Major points:

1) For this paper, the authors produced a new construct to express simultaneously the nuclear marker, the Akt and the ERK biosensors. The tree parts are connected by P2A peptides that determine their separation. Although, the biosensors are based on existing ones, the connection between them by P2A might create artifacts if the separation of the two parts is incomplete. For that, important controls are missing, such as treatment with an ERK and an Akt inhibitor. If the two parts are well separated the inhibitors should block the cytosol translocation of one of the two components and not of the other. This control is also important to check if in HeLa cells the Akt biosensors is not phosphorylated by ERK as well, as described in other reports. Alternatively, P2A separation can be quantified on a protein blot.

We agree that it is important to establish that the P2A sequence results in separation of the reporters. There are several observations that support our notion that the separation is efficient. First, we have been using the 2A-like sequences for over a decade in HeLa cells (first paper: doi:10.1038/nmeth.1415) and we have never encountered situations where the cleavage was problematic. Second, the distribution in signal of the nuclear Scarlet probe differs substantially from that of the mTurquoise2 and the mNeonGreen probe. Third, the dynamics of the ERK-KTR and Akt-KTR are different. Fourth, we have included new data with an ERK inhibitor, showing that the Akt-KTR responds independently of the ERK-KTR (figure S5). We have also added text to explain this: “Next, we examined the effect of the MEK inhibitor PD 0325901. Pre-incubation with the inhibitor for 20 minutes blocked the response of the ERK-KTR to FBS, but not that of Akt-KTR (Supplemental Figure S5). This supports previous observations [14] [15] that the P2A effectively separates the different components, since the Akt-KTR and ERK-KTR show independent relocation patterns.”

© 2022. Published by The Company of Biologists under the terms of the Creative Commons Attribution License (https://creativecommons.org/licenses/by/4.0/).

This latter point is also supported by the co-incidence plot of the ERK versus Akt clusters (figure 8C) showing that the probes act independently (which is the main reason for using this strategy). Although any of the aforementioned points cannot exclude that a small fraction of the probe remains fused, we think that this potential issue is far outweighed by the benefits of the use of 2A peptides.

2) The description of ERK and Akt should be reported in a more uniform way, such as using the same representations for both (e.g. the equivalent of figure 2 for Akt is missing) or the same number of clusters.

We choose to concentrate first on ERK activity, that is why a similar plot for Akt activation is not shown. However, the Akt responses are detailed in figure 4 and supplemental figures S5 and S7. For the cluster analysis, we looked into the optimal number of clusters (as explained in Supplemental note S2). This number differs for ERK and Akt, since the complexity of the responses is different. We move supplemental note 2 to the main text, which also clarifies the different number of clusters that we used for the analysis.

3) Figure 3 & Figure 5: It seems that the YM and YM+PTx data for the UK 14304 data is missing. This would be an interesting addition to the manuscript, and it is easy to add. A similar analysis for the Akt sensor is missing in figure 3 and should be added for consistency. Figure 4 shows data for Akt, but as timeseries and only for Histamine. See point 2, it would benefit the reader greatly if ERK and AKT are presented in a more uniform and complete fashion throughout the manuscript.

We agree that it is valuable to add data for UK with YM. This data has been added, see also reply to reviewer 1, major point 3
As for the Akt data, the response was largely similar albeit with less complexity and a lower amplitude. This is the reason to focus on ERK and this is explained in the discussion: "Therefore, the measurement of Akt does not add information. Moreover, the Akt response had a relatively poor amplitude."

4) In the results text of figure 4, the authors state that "...as shown in Figure 4C-D, which is in line with the effect of histamine on ERK.". It is unclear what the authors mean with this statement, the effects of single/double inhibition of Histamine stimulation on ERK are not quantified or discussed. Both responses can be quantified more carefully and compared.

We agree that this is poorly formulated, and we rephrase it to make it clearer: "Inhibition of Gq (figure 4C) decreases the maximum activity up to ~70%, and simultaneous inhibition of Gq and Gi causes a decrease of the responses up to ~90%, as shown in Figure 4D. These Akt amplitudes and effects of inhibitors are largely similar to those observed for ERK."

5) This paper would benefit from a mechanistic investigation. For instance, the authors could investigate the pathways that lead to the generation of the pulse of ERK and Akt. These (preliminary) results presented call for deeper investigation into the signaling pathway from Gai and Gaq to ERK and AKT, and the authors are in a great position to probe this. One simple approach is to explore the upstream pathway, such as the MAPK cascade, PI3K, RTKs by means of inhibitors.

We agree that there is much that can be done with the KTR technology. To this end, we deposit the probe and make all our data analysis methods available. We hope that others will benefit from our efforts and use the tools for mechanistic studies.

6) Since different G-proteins seem to elicit similar responses on ERK and especially for Akt, it is likely a B-arrestin / beta-gamma subunit mediated mechanism? It would be interesting to hear what the authors think of this, did they investigate/consider this possibility? E.g. Perhaps blocking RTK signaling / B-arrestin signaling would reduce heterogeneity?

We appreciate this suggestion and have added a statement to the discussion: "Based on our data, we cannot exclude that beta-arrestin or RTKs play a role in the activation of ERK and Akt. To study the role of non-classical routes to ERK activation, inhibitor studies, or probes that interrogate these processes would be useful."

© 2022. Published by The Company of Biologists under the terms of the Creative Commons Attribution License (https://creativecommons.org/licenses/by/4.0/).

7) The authors should take a serious effort to summarize the data in the figures better. Many plots that can be merged/presented in a more concise way, which would improve the readability of the manuscript greatly.

We will take care to improve the data visualization during the revision. We will address any specific points that are raised.

Minor points:

1) The authors should spell out in the legend of each figure if they are representing the absolute C/N or the normalized C/N

Thanks for pointing this out. We added this information to the legends and it is also written in the materials and methods: “data was normalized by subtracting the average of two time points prior to stimulation (usually the 5th and 6th time point) from every data point.”

2) In Figure 2 the authors should show the control with no stimulus. Also would be informative to inform the reader about the stimulation protocol used, or indicate the stimulation time and length in the figure.

We have added the no stimulus control and added the information to the legend.

3) Figure 3: This figure would benefit from a different presentation of the data, it is currently confusing. E.g. Average curves per drug condition in a single graph would present the point the authors make more clear and concise, and this single cell overview can be moved to supplements.

Our main focus is on single cell analysis and we think that the current plots convey the message in a clear and transparent fashion. It is in line with the recently proposed idea of “superplot” (https://doi.org/10.1083/jcb.202001064). We also provide scripts and data, enabling anyone to replot the data if that is desired.

4) Figure 4 legend states "CN ERK" and "ERK C/N", but is depicting only Akt responses? Only in 4c the axes are labeled, this together is very confusing.

Thanks for pointing this out. This is corrected

5) Figure 5 is missing the controls with ERK and Akt inhibitors, to show the loss of correlation between the AUC of the two

We have included data with a MEK inhibitor (new supplemental figure S5) to demonstrate the specificity of the probe and it also demonstrates that Akt can be independently activated

6) Figure 6, the presumed lack of correlation between baseline activity and response should be confirmed statistically.

We have improved the presentation of figure 6. We now show only the maximal response and how this varies between conditions. It is evident from the graphical representation that the curves are similar for the different start ratios. We feel that the use of statistics is not necessary here.

7) It seems that in S1P treated cells there is a second oscillation in ERK activity well visible in figure 2 and also in S10. Could the authors comment on that?

We add text to the discussion to address this: “We observed that activation of endogenous S1P receptors resulted in a strong, but highly heterogeneous ERK-KTR response, with two peaks in a population of cells.” and “When PTx is present, the biphasic response is abolished and the first peak of activation is reduced, suggesting that the initial response is due to Gi signaling.”

In the abstract it is unclear what authors mean with "UK".

Changed to brimonidine

© 2022. Published by The Company of Biologists under the terms of the Creative Commons Attribution License (https://creativecommons.org/licenses/by/4.0/).

9) Figure 9, it would be helpful to visually repeat the typical curve of the different clusters here, to guide the reader.

This is a good suggestion and we have added the typical curves for the different clusters to the plot.

10) The observed heterogeneity in responses might be related to different cell cycle stages, did the authors investigated/consider this possibility (e.g. with a cell cycle biosensor)?

This is a very valid comment. We do consider its importance, but we did not investigate the effects of cell cycle.

Reviewer #2 (Significance (Required)):

The paper describes with high accuracy the dynamics of ERK and Akt biosensors downstream of several GPCRs.

However, it feels like this is a preliminary report that leaves many important questions still open. It does not provide mechanistic insight and doesn't fully exploit the potential of single-cell technologies. The authors have the tools to investigate several important questions that are left open in the manuscript (e.g. connection Gaq/Gai to ERK/AKT, B-arrestin/betagamma involvement). Moreover, some important controls are missing. The authors should also consider the data presentation in the figures, to improve readability and interpretation of the manuscript.

Properly revised, would be of interest for a broad audience in cell biology, specifically GPCR and RTK signaling fields.

Expertise in cell biology, gpcr and rtk signaling, fluorescent biosensors.

Referee Cross-commenting

I agree with the assessments by the other reviewers.

Indeed showing the dynamic range of the biosensors, as Reviewer #3 states, would strengthen the manuscript and put the S1P response heterogeneity in context.

Reviewer #3 (Evidence, reproducibility and clarity (Required)):

This manuscript uses a live-cell biosensor approach to examine the activity kinetics of the ERK and Akt kinases in response to different GPCR ligands. The paper provides a detailed description of the development of a HeLa reporter cell line that expresses both Akt and ERK biosensors, along with a nuclear marker for use in cell tracking. The authors then catalog the individual responses from thousands of cells to three GPCR ligands. Individual cells show strong correlation in stimulated ERK and Akt activity. Using inhibitors for Gq and Gi proteins, it is shown that ERK and Akt activities are dependent on different G proteins. The authors also show that the heterogeneous responses within each population can be decomposed into several clusters representing similar dynamic behaviors; the frequencies of these clusters increase or decrease depending on treatments.

Overall, this is a well documented extension of an existing biosensor approach to examine GPCR signaling, and the approach is clearly described. There are however, some control experiments that are essential to support the conclusions.

Major comments:

1. The maximal responses of ERK and Akt biosensors in the selected cell clone are not adequately shown. Although FBS responsiveness is used as a validation and selection criterion, it would be much more informative to show the distribution of single-cell responses for defined activators of ERK and Akt, such as EGF and IGF-1, respectively. Without seeing the variability in these responses, it is difficult to put the heterogeneity observed in GPCR responses into context.

© 2022. Published by The Company of Biologists under the terms of the Creative Commons Attribution License (https://creativecommons.org/licenses/by/4.0/).

The FBS is used as a (crude) way to examine responsiveness of the clones. We understand that treatment of the cells with growth factors would add more data and therefore more information to the manuscript. However, the main aim of the study is to examine whether KTR technology can be used to study endogenous GPCR signaling. It is clear that the answer is positive. Next, we asked whether we could detect differences for different GPCRs and that was the focus of this study. It is unclear how studies with EGF would add new information to our observations.

2. It is not clear whether the basal activity for the biosensors represents actual activity or simply the measurement floor. This should be established by using saturating treatment inhibitors for ERK and Akt to determine the biosensor readings in the absence of any activity. Ideally, an approach such as the one shown by Ponsioen et al. (PMID: 33795873) should be used to determine the dynamic range of the sensors.

We studied the basal levels and the effect of serum. We found that the basal levels are reduced by replacing the growth medium with serum free medium. The reduction in C/N ratio reaches a plateau after ~ 2hours of replacing the medium. This data is added as supplemental figure S4. Therefore, we have performed all experiments 2 hours after replacing the growth medium with serum free imaging medium.

3. Because the biosensors are separated by self-cleaving peptides, there is the potential that incomplete cleavage could complicate the results. Cleavage efficiency should be assessed by western blot or an equivalent method.

We agree that it is important to establish that the P2A sequence results in separation of the reporters. There are several observations that support our notion that the separation is efficient. First, we have been using the 2A-like sequences for over a decade in HeLa cells (first paper: doi:10.1038/nmeth.1415) and we have never encountered situations where the cleavage was problematic. Second, the distribution in signal of the nuclear Scarlet probe differs substantially from that of the mTurquoise2 and mNeonGreen probe. Third, the dynamics of the ERK-KTR and Akt-KTR are different. Fourth, we have included new data with an ERK inhibitor, showing that the Akt-KTR responds independently of the ERK-KTR (figure S5). We have also added text to explain this: “Next, we examined the effect of the MEK inhibitor PD 0325901. Pre-incubation with the inhibitor for 20 minutes blocked the response of the ERK-KTR to FBS, but not that of Akt-KTR (Supplemental Figure S5). This supports previous observations [14] [15] that the P2A effectively separates the different components, since the Akt-KTR and ERK-KTR show independent relocation patterns.” This latter point is also supported by the co-incidence plot of the ERK versus Akt clusters (figure 8C) showing that the probes act independently (which is the main reason for using this strategy). Although any of the aforementioned points cannot exclude that a small fraction of the probe remains fused, we think that this potential issue is far outweighed by the benefits of the use of 2A peptides.

4. Ideally, an alternate method such as immunofluorescence for phosphorylated ERK/Akt or their substrates could be used in a subset of the conditions to validate the heterogeneity observed by the biosensors.

We thank the reviewer for this suggestion. Since we see a lot of variability in the dynamics, which cannot be addressed by immunofluorescence, we do not think this will experiment be valuable. Of note, GPCR activity is known to induce ERK activity in a dose-dependent manner on a population level as determined with immunolabeling methods and that is what we observe with the ERK KTR as well.

Minor comments:

1. In the introduction, more rationale and background could be provided for the examination of GPCR-stimulated ERK and Akt activity. There is not much information provided on why this is an interesting question. Other than the involvement of beta arrestin and RTK transactivation, which are mentioned, what mechanisms are known to be involved? Also, the importance of ERK and Akt in cancer is brought up, but it is not made clear how this approach or results would connect specifically to a cancer model.

© 2022. Published by The Company of Biologists under the terms of the Creative Commons Attribution License (https://creativecommons.org/licenses/by/4.0/).

We think that the connections between heterotrimeric G-proteins and kinase activity are not well established. Except for the classical Gq -> PKC -> ERK pathway, not so much is known and we add this to the discussion: “The classic downstream effector of Gq is PKC, which can activate ERK. On the other hand, it is not so clear how Gq would affect Akt. The molecular network that connects the activity of Gi with kinases also not so clear.”

2. It would be helpful to provide some explanation for why the UK+YM and UK+YM+PTx data are not shown in figure 3

We agree that this is valuable data to include. Unfortunately, this experiment was done in a slightly different condition than the other experiments (different spacing of the time intervals) and we initially skipped the data for these reasons. After careful examination of the data, we have decided to include these data (added to figures 3 & 5).
We note that we still miss the data from the YM+PTx data for UK and we have currently no way to carry out these experiments (mainly due to lack of funding). We prefer to show the YM+PTx data for the other two conditions.

3. In the Abstract figure, it is not clear which samples "Inhibitor" and "Agonist" are referring to.

Thanks for this comment. We will remove the visual abstract when the preprint is submitted to a journal.

Reviewer #3 (Significance (Required)):

While similar reporter approaches have been used in a number of papers to examine growth factor signaling dynamics of ERK and Akt, this manuscript is the first I have seen to examine the responses of these kinases to different GPCR ligands. In doing so, it adds significantly to the growing body of literature on single-cell signaling responses. The mechanisms of ERK and Akt activation by GPCRs remain somewhat ambiguous, and the data reported here will be helpful in refining models for this signal transduction process. The findings that the GPCR ligands examined show different G protein dependencies than anticipated is an interesting facet, as is the observation that, while ERK and Akt are generally correlated, inhibition of Gi preferentially blocks S1P-induced ERK activity more so than Akt activity. However, the main findings of heterogeneity in signaling, and the observation of clusters that describe the different dynamic behaviors present within a population, are highly consistent with what has been shown in other systems. Overall, this study is a useful confirmation that GPCR signaling to ERK and Akt follows a similar pattern to other forms of stimulation.

Referee Cross-commenting

Regarding the dynamic range, I don't think it is necessary to do a western blot (though this would be nice) - I think it would be sufficient to show maximal activation using EGF/IGF and full suppression using MEK/ERK and Akt inhibitors.
I also agree that all the points raised by the other reviewers. In particular, a deeper exploration and better visualization of the relationship between ERK and Akt would be very useful, as noted by both Reviewers #1 and #2.

© 2022. Published by The Company of Biologists under the terms of the Creative Commons Attribution License (https://creativecommons.org/licenses/by/4.0/).

Original submission

First decision letter

MS ID#: JOCES/2021/259685

MS TITLE: Heterogeneity and dynamics of ERK and Akt activation by G protein-coupled receptors depend on the activated heterotrimeric G proteins

AUTHORS: Sergei Chavez-Abiega, Max L.B. Gronloh, Theodorus Theodorus W.J. Gadella Jr., Frank Bruggeman, and Joachim Goedhart
ARTICLE TYPE: Research Article

Thank you for sending your manuscript to Journal of Cell Science through Review Commons.

I am happy to tell you that your manuscript has been accepted for publication in Journal of Cell Science, pending standard ethics checks.

Reviewer 1

Advance summary and potential significance to field

Copied from my report to Review Comm.
"The authors have performed highly quantitative analyses of GPCR signaling to reveal heterogenous ERK and Akt activation patterns by using kinase translocation reporters. Using a massive number of single-cell imaging data, the authors show heterogeneous responses to GPCR agonists in the absence or presence of inhibitors.
By cluster analysis, the responses of ERK and Akt were classified into eight and three patterns. This paper is clearly written with sufficient information for the reproducibility. However, the conclusion may not be necessarily supported by the provided data as described below."

Comments for the author

The authors have revised the manuscript according to the reviewers' suggestion.
The revision is limited mostly on wording.

Comments:
1. The authors refused to change the title. This reviewer is not convinced that the work here showed that the heterogeneity of ERK activation dynamics depends on the activated heterotrimeric G proteins. The authors show just one GPCR for each class. It may reflect the nature of specific GPCRs or ligands.
Furthermore, as the authors admit, the dynamic range of Akt biosensor is not large enough, rendering the analysis incomplete.
2. Fig. 4: Wrong figures are pasted here. The data are for S1P.

© 2022. Published by The Company of Biologists under the terms of the Creative Commons Attribution License (https://creativecommons.org/licenses/by/4.0/).
